# Effects of different exercise modalities on depressive symptom score changes in patients with type 2 diabetes: a systematic review and network meta-analysis

**DOI:** 10.3389/fpubh.2026.1880248

**Published:** 2026-07-08

**Authors:** Kezhi Wang, Wenjun Xu, Shouyang Cao, Yunxiao Liu

**Affiliations:** 1 School of Sports Science, Qufu Normal University, Jining, China; 2 School of Education, Beijing Sport University, Beijing, China

**Keywords:** depressive symptoms, exercise intervention, network meta-analysis, randomized controlled trial, type 2 diabetes

## Abstract

**Background:**

Type 2 diabetes mellitus (T2DM) is a major global public health challenge, and depressive symptoms are common among patients with T2DM. These symptoms may impair quality of life, self-management behaviors, glycemic control, and long-term prognosis. Exercise is a promising non-pharmacological strategy, but the comparative effects of different exercise modalities on depressive symptom scores remain unclear.

**Objective:**

This study aimed to evaluate and compare the effects of different exercise modalities on depressive symptom score changes in patients with T2DM.

**Methods:**

PubMed, Embase, Cochrane Library, Web of Science, SPORTDiscus, and MEDLINE were searched from inception to April 2026. Randomized controlled trials involving adults with T2DM were included if they compared structured exercise interventions with usual care or non-exercise controls and reported depressive symptom outcomes. Standardized mean differences (SMDs) were used because different depression scales were applied across studies. Exercise modes were classified as aerobic exercise or walking training (AE), combined aerobic/resistance or walking/resistance training (COMB), mind–body exercise (MBE), aquatic aerobic training (AT), and control (CON). Pairwise random-effects meta-analysis and frequentist network meta-analysis were performed. SUCRA values were used to describe ranking probabilities, and CINeMA was used to assess the certainty of evidence.

**Results:**

The network meta-analysis showed that, compared with the control group, MBE showed a relatively clear reduction in depressive symptom scores (SMD = −1.09, 95% CI: −2.03 to −0.14). AE, COMB, and AT also showed potential improvement trends, although some confidence intervals were wide or crossed the null line. SUCRA rankings were highest for MBE (72.4), followed by AT (69.5), COMB (69.4), AE (33.4), and CON (5.6). However, the similar SUCRA values for MBE, AT, and COMB, together with limited direct evidence and uncertainty in some comparisons, suggest that these rankings should not be interpreted as definitive clinical superiority. The overall certainty of evidence was moderate to low.

**Conclusion:**

Structured exercise interventions may improve depressive symptoms in patients with T2DM, with MBE appearing to be the most promising mode. However, these findings should be interpreted cautiously because of heterogeneity, wide confidence intervals, and limited direct evidence. More high-quality randomized controlled trials with larger samples and long-term follow-up are needed.

**Systematic review registration:**

https://www.crd.york.ac.uk/PROSPERO/view/CRD420261374583.

## 1 Introduction

Type 2 diabetes mellitus (T2DM) is a common metabolic disorder. The latest data from the International Diabetes Federation show that, in 2024, approximately 589 million adults aged 20–79 years worldwide had diabetes, and this number is expected to increase to 853 million by 2050 (1). Among global patients with diabetes, T2DM accounts for more than 90% and has become an important issue in chronic disease prevention and control and public health governance. There is a bidirectional association between diabetes and depression (2). For example, Farooqi et al. found that the prevalence of depression among patients with type 2 diabetes was approximately 19%, higher than the 11% observed in the non-diabetic population (3). Holt et al. pointed out that the prevalence of depression among patients with diabetes is approximately twice that of the general population, and that depression is associated with worsening diabetes progression, complications, and an increased risk of mortality (4). Therefore, alleviating depressive symptoms in patients with T2DM is of great significance for improving their quality of life and long-term disease prognosis.

Several existing meta-analyses have examined the effects of exercise on depressive symptoms in patients with type 2 diabetes. Narita et al. systematically evaluated the intervention effects of physical activity on diabetes-related depression and suggested that physical activity may help improve depressive symptoms in patients with diabetes; however, the included population in that study was not strictly limited to patients with type 2 diabetes (5). Mohammad et al. investigated the effects of exercise interventions on psychosocial outcomes and glycemic control in patients with type 2 diabetes based on randomized controlled trials, and the results showed that exercise could improve depression, mental health, and glycated hemoglobin levels; however, that study mainly focused on the overall effects of exercise interventions and did not further compare differences in efficacy among different exercise modes (6). Arsh et al. found that exercise could effectively alleviate depressive symptoms in patients with type 2 diabetes; however, some studies included in that review allowed the control group to receive exercise interventions, which may have confounded the study results (7). Tang et al. examined the effects of high-frequency short-duration exercise on reducing anxiety and depression in patients with type 2 diabetes, but their focus was mainly on exercise frequency and duration, and comparisons among different modes within exercise therapy remained insufficient (8). Luo et al., from the perspective of traditional mind–body exercise, explored the effects of Baduanjin on blood glucose, depression, and anxiety in patients with type 2 diabetes complicated with emotional disorders, and the results suggested that Baduanjin has certain value in mind–body regulation; however, that study focused only on a single form of exercise and could not answer the question of which exercise mode is superior among different exercise modes (9). In fact, different types of exercise do not have completely identical intervention effects, and they differ in physiological regulatory mechanisms, exercise load structures, psychological experience patterns, and patient adaptability. Therefore, simply determining whether exercise interventions may improve depressive symptom scores is no longer sufficient to meet the needs of clinical decision-making and practical application. It is more necessary to further compare the relative effects of different exercise modalities on changes in depressive symptom scale scores.

Based on this, the present study conducted a systematic review and network meta-analysis to evaluate the effects of different exercise types on changes in depressive symptom scores in patients with type 2 diabetes, and to further compare the relative effects and probability rankings among different exercise modalities. In this study, “effect” mainly refers to the between-group differences in changes in depressive symptom scale scores before and after the intervention, rather than depression remission rates, cure rates, or clinical treatment superiority in the clinical diagnostic sense. The findings may provide a reference for mental health management and individualized exercise intervention selection in patients with T2DM.

## 2 Methods

### 2.1 Registration

This systematic review and network meta-analysis protocol was registered in PROSPERO (registration number: CRD420261374583) and was conducted in accordance with the Preferred Reporting Items for Systematic Reviews and Meta-Analyses for Network Meta-Analyses (PRISMA-NMA) guidelines (10).

### 2.2 Literature search strategy

Two researchers independently managed and screened the literature using EndNote 21 (11). This study conducted a systematic electronic search in PubMed, Embase, Cochrane, Web of Science, and EBSCO (SPORTDiscus, MEDLINE). For the searches in PubMed, Cochrane, and Embase, terms from MeSH and Emtree were used, respectively. Supplementary Appendix S2 provides the search formulas for the search strategies. The search strategy was based on key phrases related to the PICOS tool: (P) Population: Diabetes Mellitus, Type 2; (I) Intervention: exercise; (C) Comparator: no exercise or usual care; (O) Outcomes: primary outcome: Depression; (S) Study type: RCTs. In addition, we searched the reference lists of selected articles and reviews to identify any relevant studies that may have been missed by the electronic search. All English-language randomized controlled trials published from database inception to April 20, 2026, were included.

### 2.3 Eligibility criteria

Studies meeting the following criteria were included: (1) randomized controlled trials; (2) adult patients (≥18 years old) diagnosed with type 2 diabetes mellitus (T2DM) according to recognized diagnostic criteria (such as the American Diabetes Association, World Health Organization, and International Diabetes Federation); (3) studies comparing an experimental group receiving a structured exercise training program for at least 6 weeks; (4) control group: no exercise or usual care; (5) depression values were assessed before and after exercise, with no restriction on the assessment questionnaire. The following studies were excluded: (1) duplicate publications; (2) literature review papers; (3) abstracts published in conference proceedings. Two researchers independently screened the articles according to the inclusion and exclusion criteria. Multiple publications from the same trial were collated, and the first or most complete report was used as the primary reference.

### 2.4 Exercise categories

For the included RCTs, we used five categories to classify the exercise interventions: 1. Aerobic or walking (AE). 2. AE combined with RT or walking combined with RT (COMB). 3. Yoga, Taichi, PET, breathing relaxation training and balance-coordination exercises (MBE). 4. Aerobic aquatic training (AT). 5. Control group: no exercise or usual care (CON).

Aerobic exercise or walking training (AE): This refers to exercise modalities primarily aimed at improving cardiorespiratory endurance or increasing aerobic activity, including walking, treadmill training, conventional aerobic exercise, land-based aerobic exercise, and high-intensity interval training (HIIT). If the main component of the intervention was continuous or intermittent aerobic activity and resistance training was not simultaneously included as a major training component, the intervention was classified as AE. Combined aerobic/resistance or walking/resistance training (COMB): This refers to exercise programs that included both aerobic exercise or walking training and resistance training, with both components constituting major parts of the intervention. For studies that also included dietary guidance, health education, or behavioral support, the intervention was classified as COMB if its core component remained the combination of aerobic/walking training and resistance training, while the potential confounding effects of non-exercise components were considered when interpreting the results. Mind–body exercise (MBE): This refers to exercise modalities that combine physical activity with breathing regulation, focused attention, postural control, mind–body relaxation, or balance and coordination, including yoga, Tai Chi, proprioceptive training, breathing relaxation training, and balance-coordination exercises. The common feature of this category is the simultaneous emphasis on physical practice and psychological regulation, although different intervention forms may vary in exercise intensity, degree of psychological engagement, supervision mode, and mechanisms of action. Aquatic aerobic training (AT): This refers to exercise modalities performed mainly in an aquatic environment and primarily involving aerobic activity, including swimming, aquatic aerobic training, and combined aquatic aerobic training. If the intervention mainly used the buoyancy and low joint-loading environment of water to conduct aerobic activity, it was classified as AT. Control group (CON): This refers to participants who did not receive structured exercise training, including no exercise, usual care, maintenance of the original lifestyle, health education, enhanced usual care, telephone communication, active immersion, or other attention-control conditions. According to the nature of the control condition, control groups were further recorded as passive controls or active/attention-control conditions in this study.

### 2.5 Data extraction

Data extraction was independently completed by two researchers and cross-checked, and any disagreements were resolved through discussion. The extracted information was as follows: primary author, publication year, country, participant characteristics (number of participants in the experimental and control groups, sex, age), intervention information (exercise type, intensity, duration, frequency, time period, supervised or unsupervised), type of control condition (no exercise, usual care, health education, enhanced usual care, active immersion, telephone communication, or other attention-control conditions), measurement methods and units of the reported outcomes. For multi-arm studies, data from each intervention arm and control arm were extracted separately, and the study ID and corresponding exercise node were recorded. In cases of insufficient information, if necessary, the authors of the included studies were contacted by email to obtain missing values.

### 2.6 Risk of bias and CINEMA assessment

Reviewers independently assessed the risk of bias within studies using the Cochrane Risk of Bias tool version 2.0 (12). The signaling questions and criteria were followed to determine the risk of bias assessment for the effect of assignment to intervention. The domains were: (1) arising from the randomization process, (2) due to deviations from intended interventions, (3) due to missing outcome data, (4) in measurement of the outcome, and (5) in selection of the reported result. Each domain was judged as having a high, moderate (some concerns), or low risk of bias, and the studies were subsequently classified for overall risk of bias as high, moderate, or low.

We used CINeMA to evaluate the confidence in the evidence for the network estimates of the primary outcome (13). The assessment was mainly based on six domains: within-study bias, reporting bias, indirectness, imprecision, heterogeneity, and incoherence. Within-study bias was judged according to the RoB 2 assessment results and the contribution of each study to the network estimates; indirectness was assessed according to the consistency of the study population, interventions, control conditions, and outcome measures with the research question; imprecision was mainly judged based on whether the 95% confidence interval crossed the null line, the prespecified clinically important effect threshold, and its width; heterogeneity was judged by considering between-study variability and prediction intervals; incoherence was used to assess the consistency between direct and indirect evidence. It should be noted that this study referred to both local and global incoherence results: loop-specific inconsistency tests and node-splitting analyses mainly reflected local incoherence in specific closed loops or specific comparisons, whereas the global inconsistency test reflected whether there were systematic differences between direct and indirect evidence at the whole-network level. When local tests did not detect significant incoherence but the global test suggested inconsistency, the incoherence domain for the relevant comparisons was downgraded accordingly in the CINeMA assessment. Finally, the certainty of evidence for each network comparison was rated as high, moderate, low, or very low.

### 2.7 Data synthesis and statistical analyses

This study preferentially used pre- and post-intervention change values to calculate effect sizes. For studies that did not directly report the standard deviation (SD) of change values, the formula provided in the Cochrane Handbook for Systematic Reviews of Interventions (Version 6.3) was used (14). The formula is:

$$\text{SDchange}=\sqrt{SD_{\text{baseline}}^{2}+SD_{\text{final}}^{2}-(2\times \text{Corr}\times SD_{\text{baseline}}\times SD_{\text{final}})}.$$

The SD of change values was calculated from the baseline SD, post-intervention SD, and the pre-post correlation coefficient. Because most studies did not report the pre-post measurement correlation coefficient, the correlation coefficient was set at *r* = 0.7 in this study. For missing SDs, conversions were preferentially performed based on the standard error (SE), 95% confidence interval, *p*-value, or *t*-value; if the data were still unavailable, the original authors were contacted to obtain the relevant data. Because different depressive symptom scales were used across the included studies, standardized mean differences (SMDs) and their 95% confidence intervals (CIs) were used as the main effect sizes. The direction of all effect sizes was standardized as follows: negative values indicated a reduction in depressive symptom scores, favoring exercise intervention; positive values indicated an increase in depressive symptom scores or insufficient improvement. A random-effects model was used to estimate the pooled effects. Because different depressive symptom scales were used across the included studies, standardized mean differences (SMDs) and their 95% confidence intervals (CIs) were used as the main effect sizes. To assist in interpreting the clinical meaning of SMDs, this study referred to commonly used effect size interpretation criteria, in which |SMD| values of approximately 0.20, 0.50, and 0.80 were regarded as small, moderate, and large effects, respectively. Meanwhile, combined with the prespecified clinically important effect threshold in the CINeMA assessment (SMD = ±0.20), if the 95% CI crossed the null line or crossed ±0.20, this indicated that uncertainty remained in the statistical certainty or clinical interpretation of the effect. Heterogeneity was assessed using the I^2^ statistic and Cochran’s Q test. When I^2^ was greater than 50% or the *p* value of the Q test was less than 0.10, significant heterogeneity was considered to exist (15). Under the random-effects model, the 95% prediction interval (95% PrI) was further calculated to assess the range within which the true intervention effect may be distributed in future similar studies. The prediction interval considers both the uncertainty of the pooled effect estimate and between-study heterogeneity, and therefore can serve as a supplementary indicator for judging the stability and generalizability of the results (16). STATA 17.0 software (StataCorp, College Station, TX, USA) was used to perform a random-effects multivariate network meta-analysis within the frequentist statistical framework to obtain pooled estimates and 95% confidence intervals (17), following the current PRISMA network meta-analysis guidelines (10). For multi-arm trials, each intervention arm was assigned to the corresponding node according to its core exercise component, and the multi-arm structure of the same study was retained in the network meta-analysis. Comparisons from the same source study were identified by study ID to avoid repeated calculation or inflated precision. Active control or attention-control conditions that did not include structured exercise training, such as health education, enhanced usual care, active immersion, or telephone communication, were uniformly assigned to the CON node, and their potential to weaken between-group differences was considered when interpreting the results. Because the included studies used different scales to measure depressive symptoms, this study used SMD as the effect size indicator for depressive symptoms. The comparative relationships among exercise interventions were presented using a network plot. In the network plot, nodes represent different types of exercise interventions, and the lines between nodes represent direct head-to-head comparisons between interventions; the node size and line thickness are proportional to the number of studies included for the corresponding intervention and direct comparison, respectively. Meanwhile, a network contribution plot was drawn to calculate the contribution of each direct comparison to the overall network estimate.

The transitivity assumption was evaluated by assessing the inclusion criteria of individual studies, judging whether all participants in the network could theoretically be randomized to receive any intervention, and using the consistency model (18). The inconsistency factor (IF) and its 95% confidence interval were used to assess the degree of consistency in each closed loop. When the lower limit of the 95% confidence interval of the IF included or was equal to 0 (19), it indicated that there was no obvious inconsistency in the closed loop. When the lower limit of the 95% confidence interval of the IF included or was equal to 0, it indicated that there was no obvious inconsistency in the closed loop. The inconsistency model was further used to test inconsistency in the network; when the inconsistency test result was not significant (*p* > 0.05), the consistency model was used for analysis (20). Meanwhile, node-splitting analysis was used to examine local inconsistency, and the results were reliable, with *p* > 0.05.

The surface under the cumulative ranking curve (SUCRA) was used to describe the probabilistic ranking of different exercise modalities within the current evidence network (21). The SUCRA score ranges from 0 to 100, with 100 indicating the best treatment effect with no uncertainty, while 0 indicates the worst treatment effect with no uncertainty (22). However, SUCRA values only reflect ranking probabilities and are not directly equivalent to the magnitude of clinical effects, nor should they be interpreted as definite clinical superiority when evidence uncertainty is high, direct comparisons are limited, or confidence intervals are wide. Based on the network meta-analysis, to further explore the potential sources of between-study heterogeneity, this study used R software (version 4.5.0) to conduct exploratory meta-regression analyses of study-level covariates (Age, Week, Frequency, Duration). To examine NMA publication bias caused by small-study effects, this study plotted a network funnel plot and made a visual judgment based on graphical symmetry. Meanwhile, Begg’s test was combined to statistically test publication bias.

### 2.8 Operational definition of the effect indicator

In this study, the “effect” of different exercise modalities on depressive symptoms was operationally defined as the standardized mean difference (SMD) in changes in depressive symptom scale scores before and after the intervention between the intervention group and the control group, or between different exercise intervention groups. Because the included studies used different scales to assess depressive symptoms, such as BDI, BDI-II, CES-D, HADS, PHQ-9, SDS, HRSD/HDRS, GDS, and GHQ-28, SMD was used to standardize the direction and dimension of effects across different scales. All effect sizes were adjusted so that negative values represented a decrease in depressive symptom scores and symptom improvement, whereas positive values represented an increase in depressive symptom scores or insufficient improvement.

It should be noted that the “effect” in this study is not equivalent to efficacy in the strict clinical sense, nor does it represent the clinical remission rate, treatment response rate, or change in diagnostic status of depression. SUCRA values were used only to describe the probabilistic ranking of different exercise modalities within the current evidence network, and cannot be interpreted alone as indicating that a certain exercise modality has definite clinical superiority or should be preferentially recommended.

## 3 Results

### 3.1 Literature selection

**Figure 1:**
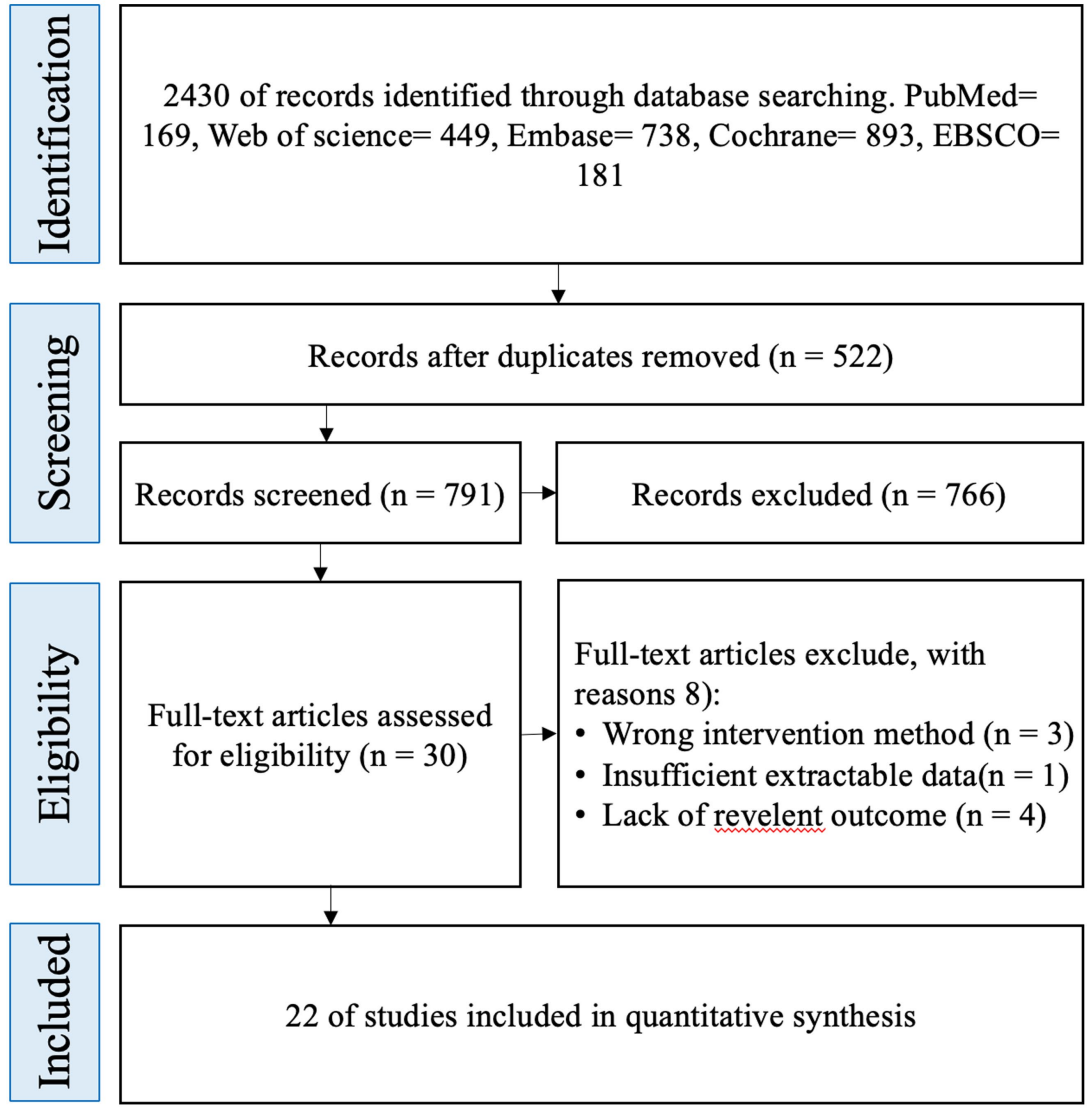
Preferred reporting items for systematic reviews and meta-analyses (PRISMA) flow diagram depicting the study selection process.

EndNote 21 software was used for literature management, and the flowchart of trial selection is shown in Figure 1. After removing 522 duplicate records, 791 articles remained for screening. By reviewing titles and abstracts, 766 articles were removed; after obtaining and reading the full texts, another 8 articles were removed, leaving 22 articles for quantitative synthesis analysis.

### 3.2 Characteristics of the included studies

This systematic review included 22 references (23–44). The characteristics of the included studies are shown in Table 1. These studies were published from 2009 (23) to 2025(44). Among them, 12 studies were conducted in Asia (23, 24, 27–29, 31, 35–38, 41, 44), including 4 from Iran (24, 27, 35, 41), 3 from Turkey (23, 32, 36), 2 from India (38, 44), 2 from Saudi Arabia (28, 36), and 1 from China (29); 3 studies were conducted in North America (25, 31, 33), all from the United States; 3 studies were conducted in Europe (26, 30, 39), from the Netherlands (26), Croatia (30), and Spain (39), respectively; 2 studies were conducted in South America (34, 42), both from Brazil; and 2 studies were conducted in Africa (40, 43), from Nigeria (40) and Egypt (43), respectively. The total sample size was 1,709, including 975 participants in the experimental groups and 734 in the control groups, with a mean age ranging from 36.35–87 years. Among them, 5 studies involved only men (24, 27, 31, 32, 35), 4 studies involved only women (33, 40, 41, 43), 13 studies included both men and women (23, 25, 26, 28–30, 34, 36–39, 42), and 1 study did not report the sex ratio (44).

**Table 1:** Characteristics of the included studies.

**Study**	**Country**	***N***	**age** **(mean[SD])**	**Type of exercise**	**Training plan**	**Period and frequency** **(week × [times/week])**	**Duration**	**Whether supervised**	**Outcomes**	**Measurement method**
Kucukarslan et al. (23)	Turkey	18 (15 M/3F)	51.39 (2.02)	RE + Walking	Resistance training + home-based walking	8 × 2	15–45 min	supervised	Depression	Depression: CES-D
		18 (12 M/6F)	56.06 (1.48)	CON(A)	Maintain regular physical activities					
Saiiari et al. (24)	Iran	30 M	36.5	Swimming	Keep low to moderate intensity	4 × 3 + 4 × 4	30 min	Unclear	Depression	Depression: BDI
		30 M		CON(P)	No exercise					
Piette et al. (25)	US	145 (71 M/74F)	55.1 (9.4)	Walking	CBT program and pedometer-based walking program	12 × 1 + 9 moth×1	45 min	supervised	Depression	Depression: BDI
		146 (73 M/73F)	56.0 (10.9)	CON(A)	Enhanced usual care					
Snel et al. (26)	Netherlands	13 (8 M/5F)	53 (3)	AE	Aerobic exercise and on a cyclo-ergometer	16 × [1 supervised + ≥ 4 home/week]	60 min	supervised	Depression	Depression: HADS
		14 (6 M/8F)	56 (2)	CON(P)	No exercise					
Sardar et al. (27)	Iran	27 M	44.93 (5.35)	AE	Ergo meter bikes	8 × 3	45–60 min	supervised	Depression	Depression: GHQ-28
		26 M	45.56 (5.41)	CON(P)	No exercise	8				
Osama et al. (28)	Arabia	50 (28 M/22F)	36.35 (5.11)	AE	Moderate intensity aerobic exercise sessions on a treadmill	3moth × (3/week)	40 min	supervised	Depression	Depression: BDI
		50 (28 M/22F)	37.16 (4.32)	CON(P)	No exercise					
Zheng et al. (29)	China	55 (27 M/28F)	62 (6)	Tai Chi	Twenty-four Move Shadow Boxing and psychosomatic relaxation	24 × 3–5 (2/day)	40 min	Partly supervised	Depression	Depression: SDS
		57 (27 M/30F)	61 (7)	CON(A)	Received community diabetes health instructions		30 min			
Pibernik-Okanovic et al. (30)	Croatia	58 (25/M33F)	58.5 (4.8)	AE	Compound aerobic exercise	6 × 1	90 min	supervised	Depression	Depression: CES-D
		57 (27 M/30F)	58.2 (5.6)	CON(A)	Enhanced treatment as usual	6 × 1	90 min			
Leehey et al. (31)	US	14 M	65.4 (8.7)	AE + RT	Aerobic and resistance exercise plus diet	12 × [3/week] + 40 × [3/week or 6/week]	30–90 min	supervised	Depression	Depression: CES-D
		18 M	66.6 (7.5)	CON(A)	Dietary management only					
Yucel et al. (32)	Turkey	24 M	58.50 (7.00)	Yoga	Pilates-based mat exercise	12 × 3	45–70 min	supervised	Depression	Depression: HADS
		21 M	53.50 (9.00)	CON(P)	No exercise					
Schneider et al. (33)	US	15F	53.3 (6.0)	EX	Different moderate intensity exercise	16 × 2 + 4 × 1 + 2 × 1	90 min	supervised	Depression	Depression: HRSD and BDI-II
		14F	53.6 (8.4)	CON(A)	Telephone communication					
Delevatti et al. (34)	Brazil	17 (7 M/10F)	54.2 (8.3)	AT	Aquatic aerobic training	12 × 3	45 min	supervised	Depression	Depression: BDI
		18 (8 M/10F)	59.2 (6.9)	DLT	Dry-land aerobic training	12 × 4	46 min			
Gilani et al. (35)	Iran	30 M	48.86 (5.76)	AE	Moderate-intensity aerobic exercise	12 × 3	45–60 min	supervised	Depression	Depression: GHQ-28
		30 M	49.08 (6.09)	CON(P)	No exercise					
Duruturk et al. (36)	Turkey	23 (12 M/11F)	52.82 (11.86)	CE	Performed breathing and callisthenic exercises at home by internet based video conferences	6 × 3	20–45 min	supervised	Depression	Depression: BDI
		21 (14 M/7F)	53.04 (10.45)	CON(A)	Received the education session					
Abdelbasset et al. (37)	Arabia	14 (8 M/6F)	53.4 (5.3)	PET	Balance- and lower-limb proprioceptive training	2 months × 3/week	45 min	supervised	Depression	Depression: HDRS
		14 (7 M/7F)	52.8 (5.7)	CON(P)	No exercise					
Singh et al. (38)	India	101 (41 M/60F)	50.3 (9.1)	Yoga	Warm-up, standing asanas, sitting asanas, prone, supine and pranayama	3 moth × (7/week)	48 min	Partly supervised	Depression	Depression: BDI
		99 (49 M/50F)	49.4 (8.7)	RE + Walking	Brisk walking and resistance training with elastic band	3moth × (5/week)	30 min			
Martínez et al. (39)	Spain	54 (29/M/25F)	86 (5)	CT	Progressive resistance, balance, walking exercise, and Functional low-intensity training	12 × 5–7	40 min	Partly supervised	Depression	Depression: GDS
		49 (21 M/28F)	87 (4)	CON(P)	Usual care					
Maharaj et al. (40)	Nigeria	25F	40.4 (7.22)	Walking	Treadmill walking	12 × 3	45 min	supervised	Depression	Depression: BDI-II
		24F	39.9 (5.77)	CON(A)	Diet, diabetes and mental health					
Donyaei et al. (41)	Iran	17F	61.3 (5.7)	AE + RT	Performed a combined resistance and endurance training program	12 × 3	60 min	supervised	Depression	Depression: BDI
		17F	62.1 (5.1)	CON(P)	No exercise					
Delevatti et al. (42)	Brazil	19 (10 M/9F)	57.5 (7.4)	AERO	Aerobic aquatic training	15 × 3	56 min	supervised	Depression	Depression: PHQ-9
		19 (9 M/10F)	60.9 (7.4)	COMB	Combined aquatic and resistance training	15 × 3	56 min			
		19 (9 M/10F)	58.6 (9.7)	CON(A)	Active control in immersion					
Elgayar et al. (43)	Egypt	37F	48.29 (5.65)	HIIT	Progressive HIIT in the form of running on an electrical treadmill.	12 × 3	22–30 min	supervised	Depression	Depression: BDI-II
		39F	50.2 (4.35)	CON(P)	No exercise					
Subramani et al. (44)	India	53	53 (7.5)	Yoga	Warm-up, asanas, nadishodhana and sitali	12 × (1/2 week)	35 min	Partly supervised	Depression	Depression: PHQ-9
		70		CON(P)	Usual care					

M: male, F: female, AE: aerobic exercise, RT: resistance exercise, CON: control, EX: different moderate intensity exercise, AT: Aquatic aerobic training, DLT: Dry-land aerobic training, CE: breathing and callisthenic exercises, PET: balance- and lower-limb proprioceptive training, CT: resistance, balance, walking exercise, and Functional low-intensity training, HIIT: high-intensity interval training, P: Passive control, A: active control.

They were combined into four categories: “AE, COMB, MBE, and AT.” Among them, aerobic exercise included walking (25, 40), conventional aerobic training (26–28, 30, 35), land-based aerobic training (34), HIIT (43) etc., with a total of 10 intervention groups and 418 participants included; combined exercise included resistance combined with walking (23), aerobic combined with resistance (31, 41) etc., with a total of 6 intervention groups and 221 participants included; mind–body exercise included yoga (32, 38, 44), Tai Chi (29), proprioceptive training (37) etc., with a total of 6 intervention groups and 270 participants included; aquatic aerobic exercise included swimming (24), aquatic aerobic training (42), and aquatic comprehensive aerobic training (42) etc., with a total of 3 intervention groups and 66 participants included.

In terms of intervention duration, the duration of exercise interventions was mainly concentrated between 6 and 24 weeks, with the longest being 52 weeks (31). Among them, 12-week (32, 34, 35, 39, 40, 43, 44) interventions were the most common, accounting for about half of all exercise intervention durations. Specifically, the intervention durations included 6 weeks (30, 36), 8 weeks (23, 24, 27), 12 weeks (32, 34, 35, 39, 40, 43, 44), 15 weeks (31, 42), 16 weeks (26), 22 weeks (33), 24 weeks (29), and 52 weeks (31). The weekly exercise frequency was mostly 3 times per week (27, 28, 32, 34–37, 40–43), and some studies set it as 1 time per week (25, 30, 38), 2 times (23), 4 times (34), or 5–7 times (39). The duration of each exercise session was mostly concentrated between 40 and 60 min (26, 27, 34, 35, 37, 40–42), ranging from approximately 15–150 min, with individual studies reporting exercise volume in the form of a weekly accumulated 150 min (38).

Depression measurement tools included BDI, BDI-II, CES-D, HADS, GHQ-28, SDS, HRSD/HDRS, GDS, and PHQ-9. Among them, BDI was used most frequently, in a total of 7 studies (24, 25, 28, 34, 36, 38, 41); CES-D was used in 3 studies (23, 30, 31); HADS (32, 37), GHQ-28 (27, 35), BDI-II (40, 43), and PHQ-9 (42, 44) were each used in 2 studies; SDS (29), HRSD/BDI-II (33), HDRS (26), and GDS (39) were each used in 1 study.

### 3.3 Risk of bias

**Figure 2:**
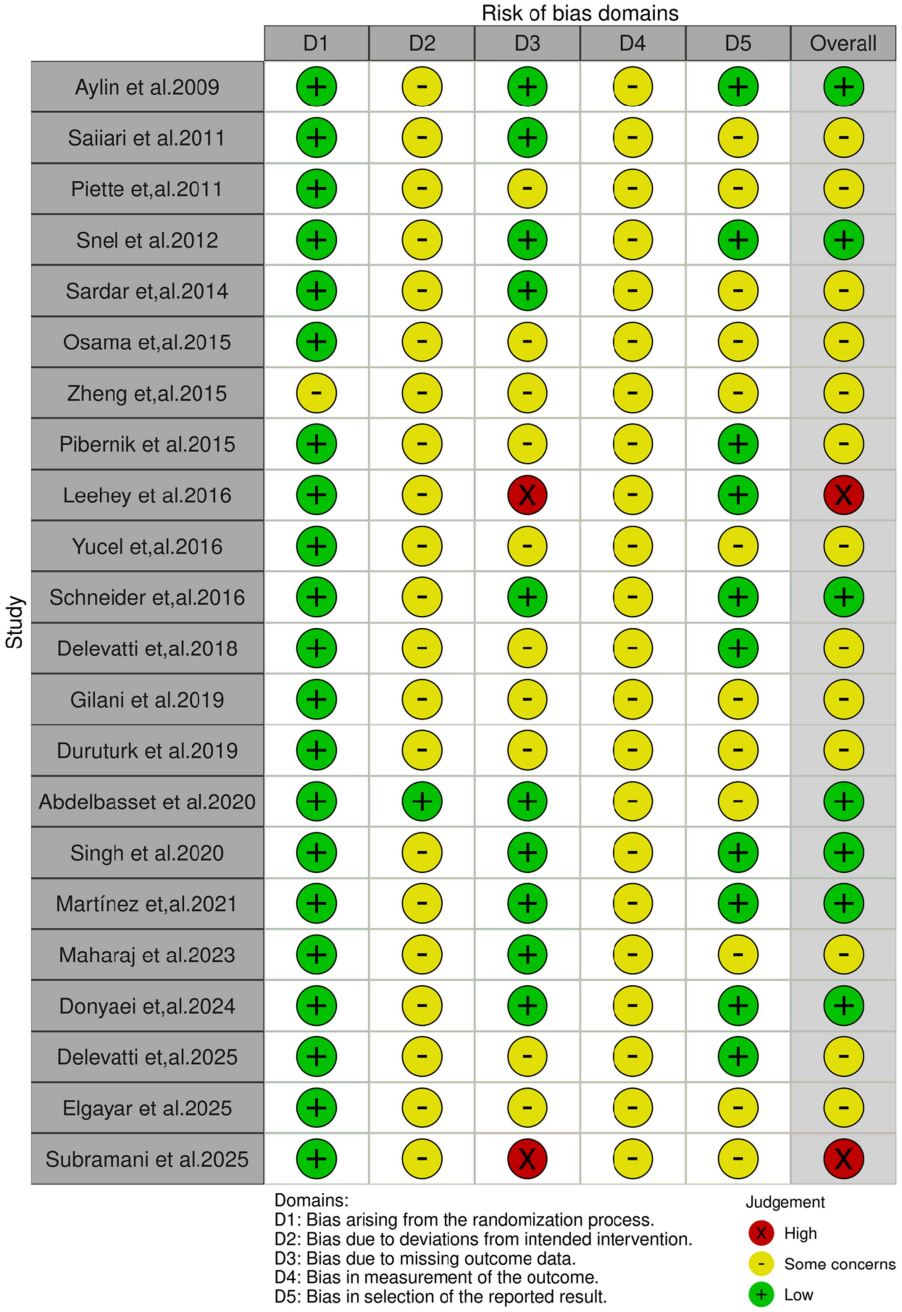
The detail of risk of bias.

**Figure 3:**
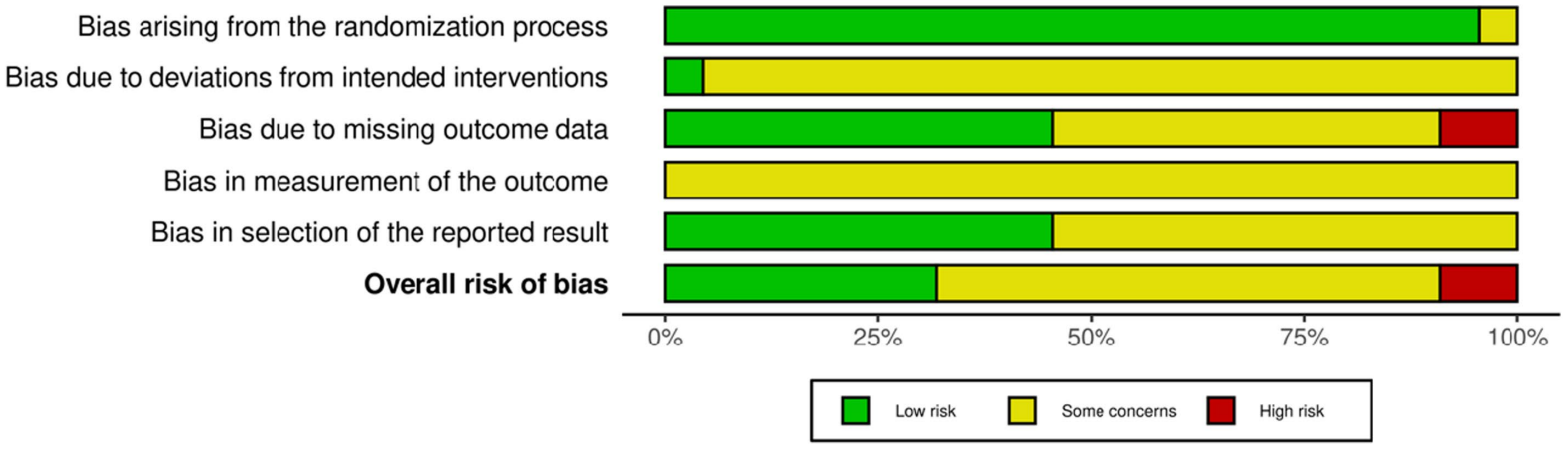
The summary of risk of bias.

Details of the RoB assessment for each study are shown in Figures 2, 3. A total of 22 randomized controlled trials were included in this study for RoB 2 risk of bias assessment. The results showed that most studies had a low risk of bias in the randomization process (D1), indicating that most studies reported random allocation methods or that baseline characteristics between groups were relatively balanced. In contrast, in the domain of deviations from intended interventions (D2), only a few studies were rated as low risk, while most studies had “some concerns.” This was mainly because exercise interventions are behavioral interventions, and it is difficult to achieve complete blinding of participants and intervention providers, which may lead to expectation effects or behavioral changes. In terms of missing outcome data (D3), about half of the studies were rated as low risk, but some studies had certain concerns due to high dropout rates, imbalanced dropout between groups, or failure to clearly use intention-to-treat analysis. Among them, 2 studies were rated as high risk, mainly because loss to follow-up or withdrawal in these studies did not occur randomly but was concentrated in the intervention group, and the dropout proportion was relatively high. In terms of measurement of the outcome (D4), all studies had some concerns, mainly because depressive outcomes were mostly assessed using subjective scales such as BDI, PHQ-9, HADS, and PSQI, and when participants could not be blinded, the results may have been affected by self-reporting bias and expectation effects. For selection of the reported result (D5), some studies had certain concerns because they did not provide trial registration information or a prespecified study protocol.

Overall, approximately 31.8% (7/22) of the included studies were rated as having a low risk of bias, approximately 59.1% (13/22) had some concerns, and approximately 9.1% (2/22) were rated as having a high risk of bias. Overall, the methodological quality of the included studies was moderate, with the main sources of bias concentrated in the inability to blind exercise interventions, the measurement of subjective psychological outcomes, and missing outcome data in some studies.

### 3.4 Network meta-analysis

**Figure 4:**
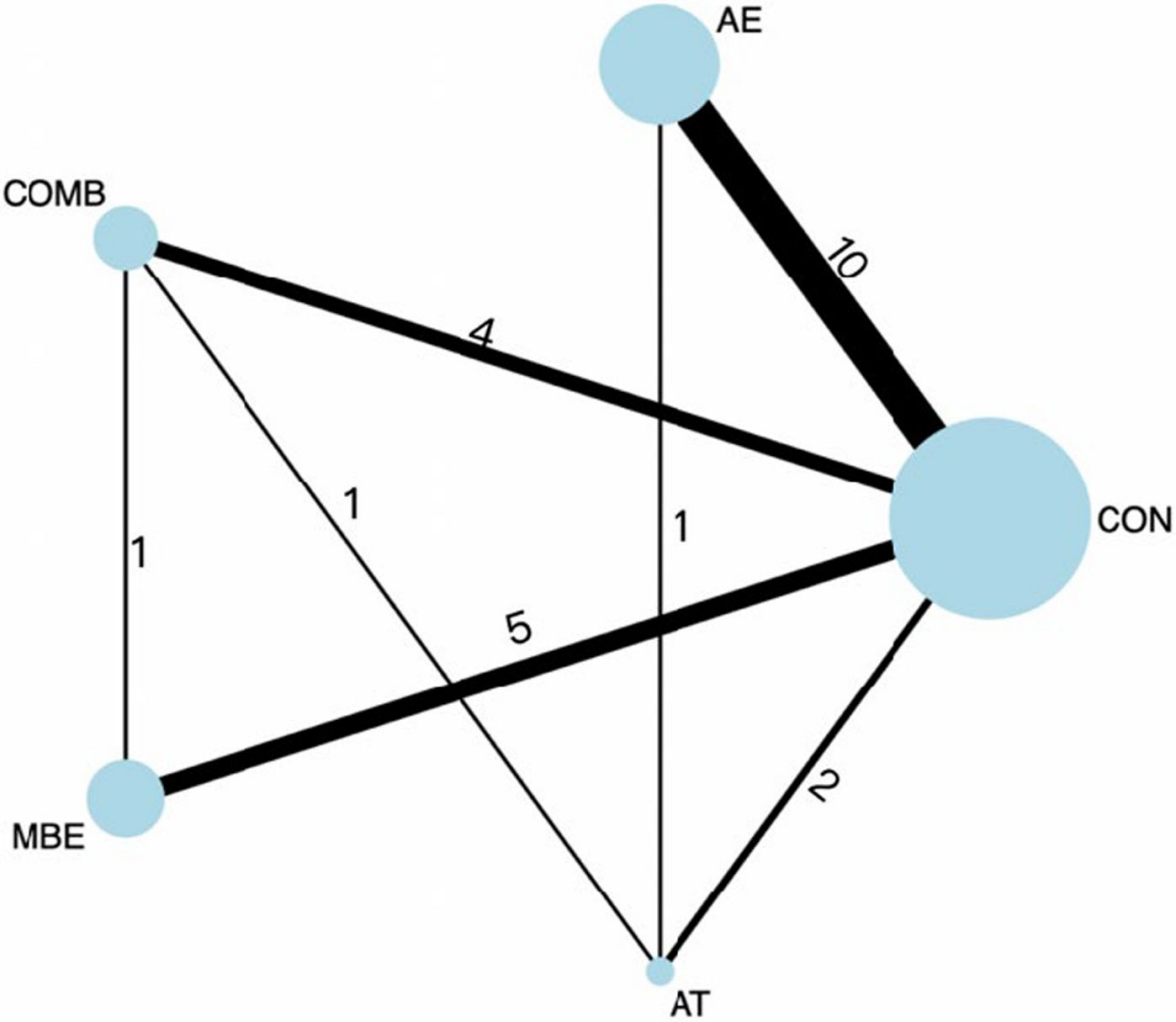
This network diagram illustrates the impact of different exercise types on the depressive symptoms of patients with type 2 diabetes.

We conducted an NMA on depression indicators to explore the effects of different exercise categories on depression, thereby verifying the effectiveness of exercise interventions. Figure 4 shows the NMA plot of the eligible studies that investigated the effectiveness of exercise categories on depressive symptoms in patients with type 2 diabetes. The size of the nodes is related to the sample size of that exercise category, and the thickness of the lines between exercise modes is related to the number of studies involving that comparison. The most common intervention type was AE, and the least common was AT. Supplementary Appendix S3 shows the contribution of direct and indirect comparisons to the NMA, as well as the number of studies for each direct comparison.

Inconsistency for depression was examined through loop-specific heterogeneity assessment, the inconsistency model, and node-splitting analysis (Supplementary Appendix S4). The results of the loop-specific inconsistency test showed that the IF values for the three closed loops CON-COMB-AT, CON-COMB-MBE, and CON-AE-AT were 2.222, 1.932, and 1.554, respectively, with *p* values of 0.486, 0.323, and 0.194, respectively, all greater than 0.05, and the 95% CIs all included 0, suggesting that no significant local inconsistency was detected in the above closed loops. The node-splitting analysis also found no significant differences between direct and indirect evidence in specific comparisons. However, the lack of statistical significance in local inconsistency tests does not mean that inconsistency is completely absent from the entire network. Further global inconsistency testing suggested that there may be systematic differences between direct and indirect evidence at the network level.

### 3.5 Pooled estimates of primary outcomes

The pooled estimates of the NMA are shown in Table 2. Compared with the control group, MBE showed a relatively clear signal of improvement in reducing depressive symptom scores (SMD = −1.09, 95% CI: −2.03 to −0.14). AE (SMD = −0.41, 95% CI: −1.11 to 0.29), COMB (SMD = −1.05, 95% CI: −2.11 to 0.02), and AT (SMD = −1.07, 95% CI: −2.36 to 0.22) also showed a trend toward reduced scores, but their confidence intervals were wide or crossed the null line; therefore, these findings cannot be used to determine definite statistical advantage or clinical superiority. The forest plots comparing each exercise type, including 95% CI and 95% prediction interval (95% Prl), are shown in Supplementary Appendix S5.

**Table 2:** Network meta-analysis matrix.

AE	−0.64 (−1.89,0.62)	−0.68 (−1.85,0.49)	−0.66 (−2.03,0.70)	0.41(−0.29,1.11)
0.64 (−0.62,1.89)	COMB	−0.04 (−1.32,1.24)	−0.03(−1.56,1.51)	1.05 (−0.02,2.11)
0.68 (−0.49,1.85)	0.04 (1.24,1.32)	MBE	0.02(−1.56,1.59)	1.09 (0.14,2.03)
0.66 (−0.70,2.03)	0.03 (1.51,1.56)	−0.02 (−1.59,1.56)	AT	1.07 (−0.22,2.36)
−0.41 (−1.11,0.29)	−1.05 (−2.11,0.02)	−1.09 (−2.03,-0.14)	−1.07(−2.36,0.22)	CON

### 3.6 SUCRA probability ranking

The SUCRA probability ranking results (Table 3) showed that MBE had the highest ranking probability (SUCRA = 72.4), followed by AT (69.5), COMB (69.4), AE (33.4), and CON (5.6). However, the SUCRA values of MBE, AT, and COMB were relatively close, and some comparisons involving AT and COMB had limited direct evidence and wide confidence intervals. Therefore, the SUCRA ranking only suggests that MBE had a higher ranking probability in the current evidence network, and should not be interpreted as indicating definite clinical superiority. Nor should it be used to directly conclude that AE is the exercise modality with the poorest clinical effect (Table 3 and Supplementary Appendix S6).

**Table 3:** Ranking of the effectiveness of exercise interventions.

Treatment	SUCRA
MBE	72.4
AT	69.5
COMB	69.4
AE	33.4
CON	5.6

### 3.7 Meta-regression analysis

**Figure 5:**
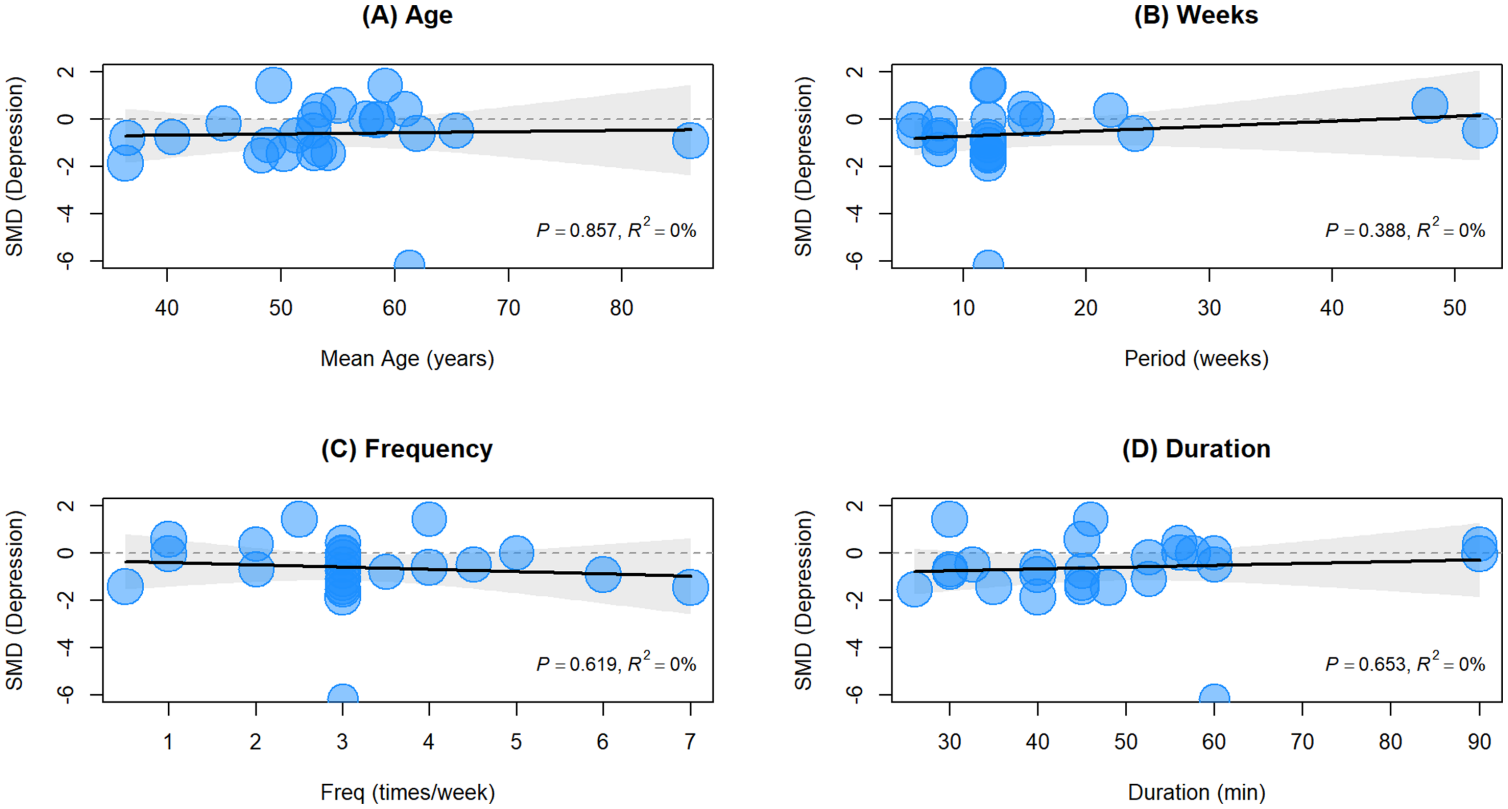
Meta-regression analysis of the effects of age and exercise dose factors on the improvement of depressive symptoms.

The meta-regression results (Figure 5) showed that mean age, total intervention duration, weekly exercise frequency, and single-session exercise duration did not significantly moderate the effect of exercise intervention on depressive symptoms. The regression slopes of the four covariates did not reach statistical significance, and the proportion of heterogeneity explained was 0%: mean age (*p* = 0.857, *R*^2^ = 0%), intervention duration (*p* = 0.388, *R*^2^ = 0%), exercise frequency (*p* = 0.619, *R*^2^ = 0%), and single-session exercise duration (*p* = 0.653, *R*^2^ = 0%).

### 3.8 Publication bias

**Figure 6:**
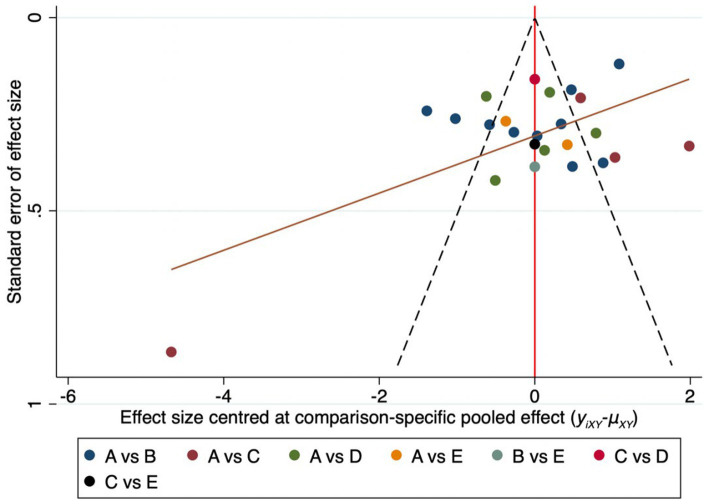
The funnel plot graphics in NMA. A-CON, B-AE, C-COMB, D-MBE, E-AT.

The funnel plot (Figure 6) showed that the scatter distribution was not completely symmetrical, and there was an obvious outlier in the lower left, suggesting that there may be some small-study effects or publication bias. Because the standard error of this outlier was large, it may have come from a study with a small sample size and a large effect size. Subsequent sensitivity analysis should be combined to further judge its influence on the pooled effect and intervention ranking results. Overall, this study did not find serious publication bias, but the influence of small-study effects on the stability of the results still cannot be completely excluded.

### 3.9 Quality of evidence assessment

Table 4 presents the CINEMA assessment results for each comparison. Overall, the quality of evidence of the included studies was mainly moderate to low. Specifically, in terms of within-study bias, some comparisons were rated as “some concerns,” mainly because a certain proportion of studies with moderate or high risk of bias among the included studies contributed to the corresponding network estimates, but overall did not reach a serious level of bias. In terms of imprecision, some comparisons turned yellow or red, mainly because their 95% CIs were wide and crossed the prespecified clinically important effect threshold, suggesting insufficient stability of the effect estimates. In terms of heterogeneity, some comparisons were rated as “some concerns” or “major concerns,” mainly related to the large between-study variance in the network meta-analysis, indicating that there may be differences in intervention protocols, sample characteristics, or outcome measurements among different studies, thereby leading to widened prediction intervals. Regarding incoherence, the loop-specific inconsistency test and node-splitting analysis did not detect significant local inconsistency, but the global inconsistency test suggested inconsistency at the network level (*p* = 0.000). Therefore, in the CINeMA assessment, the incoherence domain was downgraded for some comparisons, and caution was maintained when interpreting the network estimates and SUCRA rankings.

**Table 4:** CINEMA assessment results.

Comparison	**Number of studies**	**Within-study bias**	**Reporting bias**	**Indirectness**	**Imprecision**	**Heterogeneity**	**Incoherence**	**Confidence rating**
Mixed evidence								
CON: AE	10	Some concerns^1^	Low risk	No concerns	Some concerns^2^	Some concerns^3^	No concerns	Moderate
CON: COMB	4	No concerns	Low risk	No concerns	No concerns	Major concerns^3^	No concerns	Moderate
CON: MBE	5	No concerns	Low risk	No concerns	No concerns	Major concerns^3^	Some concerns^4^	Low
CON: AT	2	Some concerns^1^	Low risk	No concerns	No concerns	Major concerns^3^	Major concerns^4^	Low
AE: AT	1	Some concerns^1^	Low risk	No concerns	Major concerns^2^	No concerns	No concerns	Moderate
COMB: MBE	1	No concerns	Low risk	No concerns	Major concerns^2^	No concerns	No concerns	Moderate
COMB: AT	1	Some concerns^1^	Low risk	No concerns	Major concerns^2^	No concerns	No concerns	Moderate
Indirect evidence								
AE: COMB	0	Some concerns^1^	Low risk	No concerns	Major concerns^2^	No concerns	Major concerns^4^	Low
AE: MBE	0	Some concerns^1^	Low risk	No concerns	Major concerns^2^	No concerns	Major concerns^4^	Low
MBE: AT	0	Some concerns^1^	Low risk	No concerns	Major concerns^2^	No concerns	Major concerns^4^	Low

1. Evaluated based on the RoB 2.0 results and their contribution proportions to the network estimates. 2. The 95% CI crossed the prespecified clinically important effect threshold (SMD = ±0.20). 3. The between-study variance was large (*τ*^2^ = 0.906). 4. The global inconsistency test was significant (*P* = 0.000). The colors indicate the CINeMA judgment levels: green indicates no concerns, yellow indicates some concerns, and red indicates major concerns.

## 4 Discussion

### 4.1 Study summary

The current systematic review and network meta-analysis included 22 randomized controlled trials involving 1,709 patients with type 2 diabetes, and compared the effects of different exercise modalities on changes in depressive symptom scores. The results showed that, compared with the control group, MBE showed a relatively clear signal of improvement in reducing depressive symptom scores and ranked highly in the SUCRA probability ranking. This finding suggests that mind–body exercises, such as yoga, Tai Chi, proprioceptive training, breathing relaxation, and balance-coordination exercises, may have certain potential value in improving depressive symptom scores in patients with T2DM (45). However, it should be emphasized that this study evaluated changes in depressive symptom scale scores, rather than depression remission, cure, or treatment response in the clinical diagnostic sense. Therefore, the higher ranking of MBE should not be directly interpreted as indicating definite clinical superiority or as suggesting that it should be the only preferred exercise modality.

### 4.2 Research comparison and mechanistic analysis

The findings of this study are generally consistent with previous meta-analyses, indicating that physical activity can reduce diabetes-related depressive symptoms^7^. Among them, Tai Chi, yoga, Qigong, etc., can improve anxiety and depression (46). Meanwhile, walking, jogging, and strength training also show relatively prominent effects (47, 48), which is consistent with the ranking result of MBE in this study. Different from previous studies, this study focused on patients with T2DM and included MBE, AE, COMB, and AT in the same evidence network for comparison. Therefore, it not only verified the overall benefits of exercise intervention but also further suggested that different exercise modes may have differential effects on the improvement of depressive symptoms in patients with diabetes. This study had high heterogeneity, which may be related to the following factors. First, the included participants differed in age, sex ratio, diabetes duration, baseline depression severity, degree of obesity, comorbid conditions, and medication use, all of which may affect exercise adherence and the magnitude of psychological improvement. Second, there were large within-category differences among different exercise types. For example, AE included walking, treadmill training, conventional aerobic training, and possibly HIIT of different intensities; MBE included yoga, Tai Chi, breathing training, relaxation training, and balance-coordination training, and their exercise intensity, degree of psychological engagement, and supervision methods were not completely consistent. Third, intervention duration, weekly frequency, single-session duration, whether the intervention was supervised, type of control group, and whether health education or dietary guidance was also received may all lead to fluctuations in effect size. Finally, the measurement tools for depressive outcomes were not completely consistent. For example, BDI, HADS, CES-D, or other psychological scales differ in item settings, scoring direction, and sensitivity, which may also increase statistical heterogeneity.

From the perspective of mechanisms of action, the improvement of depressive symptoms in patients with T2DM through exercise intervention may not be caused by a single pathway, but rather by the combined effects of metabolic, neurobiological, and psychological behavioral mechanisms. For patients with T2DM, regular exercise can improve glycemic control, insulin sensitivity, physical function, and weight management, thereby reducing the sense of disease burden and enhancing self-efficacy and sense of control over life; the joint position statement of the ACSM and ADA also emphasizes that both aerobic exercise and resistance training can improve insulin action and contribute to T2DM management (49). At the neurobiological level, exercise may exert antidepressant effects by regulating inflammatory factors, improving circadian rhythm and sleep, increasing endorphins and monoamine neurotransmitters, increasing BDNF levels, and promoting neuroplasticity (50). MBE performed relatively prominently in this study, possibly because it not only provides physical activity stimulation but also emphasizes breath control, attention concentration, and emotion regulation, making it more likely to act on depression-related aspects such as stress response, sleep quality, and negative thinking. In contrast, AE is easier to promote and has lower cost, making it a basic form of exercise management for T2DM; COMB combines the advantages of aerobic and resistance training and can theoretically improve cardiorespiratory fitness, muscle strength, and metabolic status simultaneously; AT, due to its low impact, low joint load, and high comfort, may be more suitable for patients with obesity, joint pain, or high fear of exercise.

### 4.3 Limitations

In the current study, limitations are inevitable. First, the included studies differed in exercise types and control conditions, and some comparisons had limited direct evidence and wide confidence intervals; therefore, conclusions regarding the relative effects of different exercise modalities should still be interpreted with caution. Because the number of studies in some exercise nodes was small, further exclusion of multicomponent interventions or subgroup analyses according to control type might affect network connectivity; therefore, these sensitivity analyses were not conducted in this study. Second, the number of studies included in some exercise categories was small, especially for AT and direct comparison evidence between some exercise modes was limited, resulting in wide confidence intervals for network estimates and certain uncertainty in the ranking results. Third, the depression scales used in the included studies were not completely consistent, including BDI, CES-D, HADS, PHQ-9 etc. Although the use of SMD for pooled analysis can reduce the impact of scale differences to a certain extent, different scales may still differ in measurement dimensions, sensitivity, and clinical interpretation. Fourth, the classification of exercise interventions may have introduced some uncertainty. Although this study classified interventions according to core exercise components, training goals, and implementation forms, differences in dose, intensity, supervision mode, and intervention setting still existed within each node. Some studies also included non-exercise components such as dietary guidance and health education, which may have caused confounding. Fifth, the risk of bias in the included studies may affect the credibility of the results. Because complete blinding is difficult to achieve in exercise interventions, and depressive symptoms were mostly measured using self-report scales, the results may have been influenced by expectation effects, attention, motivation, and participants’ awareness of group allocation. Sixth, the structure of the network meta-analysis in this study still had some fragility. The number of studies included in some exercise nodes was small, and direct head-to-head comparisons between some exercise modalities were lacking, with the relevant estimates mainly relying on indirect evidence. Seventh, this study mainly included English-language literature, which may lead to language bias; meanwhile, most studies had short follow-up periods and lacked systematic evaluation of the long-term maintenance effects, adherence, and safety of exercise interventions.

### 4.4 Practical implications

This study provides practical reference for non-pharmacological interventions for depressive symptoms in patients with type 2 diabetes. The results suggest that MBE showed a relatively clear signal of score improvement in the current evidence, but uncertainty remains regarding its relative advantage. Therefore, when developing exercise intervention programs, clinical healthcare professionals and community health managers should not select exercise modalities solely based on SUCRA rankings, but should comprehensively consider factors such as patients’ baseline depression severity, physical function, comorbidities, exercise adherence, safety, risk of adverse events, exercise preferences, and resource availability. COMB, AT, and AE also have certain application value and can be selected individually according to patients’ age, degree of obesity, joint load, cardiorespiratory function, and personal preferences.

## 5 Conclusion

The results of the current systematic review and network meta-analysis showed that structured exercise interventions may help reduce depressive symptom scores in patients with type 2 diabetes. Among them, mind–body exercise (MBE) showed a relatively clear signal of score improvement compared with the control group and ranked highly in the SUCRA probability ranking. In this study, MBE included different types of interventions, such as yoga, Tai Chi, proprioceptive training, breathing relaxation training, and balance-coordination exercises. Although these intervention forms all have the characteristics of mind–body integration, they are not completely consistent in terms of exercise intensity, intervention content, supervision mode, and degree of psychological engagement. Therefore, the MBE-related results should be understood as the overall trend of this type of exercise modality within the current evidence network, and cannot be directly inferred to mean that any specific form of mind–body exercise has the same effect. It should be emphasized that the primary outcome evaluated in this study was changes in depressive symptom scale scores, rather than depression remission rates, treatment response rates, or clinical cure in the clinical diagnostic sense. Therefore, the higher ranking of MBE cannot be directly interpreted as indicating definite clinical superiority, nor should it be described as the only “best” or “preferred” exercise intervention. COMB, AT, and AE also showed certain trends of score improvement, but some comparisons had wide confidence intervals or crossed the null line, and direct evidence was limited; therefore, the stability of these results should still be interpreted with caution. The meta-regression results showed that mean age, intervention duration, weekly exercise frequency, and single-session exercise duration did not significantly affect the effect of exercise intervention, indicating that this study did not find a clear moderating effect of age or exercise dose. The evidence grading assessment showed that some comparisons were downgraded due to issues such as within-study bias, imprecision, heterogeneity, and incoherence, and the overall quality of evidence was still mainly moderate to low. However, because the ranking probabilities of different exercise modes were relatively close, and some evidence had issues such as high heterogeneity and insufficient direct evidence, more high-quality randomized controlled trials with larger sample sizes and long-term follow-up are still needed in the future to further compare the effects of different exercise modalities on changes in depressive symptom scores, clinical response, adherence, safety, and patient preferences, so as to clarify their practical application value in mental health management for patients with T2DM.

## Data Availability

The original contributions presented in the study are included in the article/Supplementary material, further inquiries can be directed to the corresponding author/s.
