# Supplementary material for: Effects of different exercise modalities on depressive symptom score changes in patients with type 2 diabetes: a systematic review and network meta-analysis
Source: Front Public Health. 2026 Jul 8;14:1880248. doi: 10.3389/fpubh.2026.1880248 (PMC13393424; doi:10.3389/fpubh.2026.1880248)
Supplement: Supplementary file 1 [file Table_1.DOCX]

**Data source：**

| **study** | type | time | frequency | duration | age | Exp Mean | Exp SD | Exp n | Cotr Mean | Cotr SD | Cotr n |
| --- | --- | --- | --- | --- | --- | --- | --- | --- | --- | --- | --- |
| **Aylin 2009** | 3 | 8 | 2 | 30.0 | 53.73 | -5.23 | 5.73 | 18 | -1.17 | 6.25 | 18 |
| ***Saiiari 2011*** | 4 | 8 | 4 | 30.0 | 36.5 | -4.77 | 6.68 | 30 | -0.06 | 5.43 | 30 |
| **Piette 2011** | 1 | 48 | 1 | 45.0 | 55.55 | 0.20 | 7.11 | 145 | -4.40 | 8.14 | 146 |
| ***Snel 2012*** | 1 | 16 | 5 | 60.0 | 54.5 | -2.00 | 1.00 | 13 | -2.00 | 0.77 | 14 |
| **Sardar 2014** | 1 | 8 | 3 | 52.5 | 45.26 | 0.11 | 0.43 | 27 | 0.19 | 0.64 | 26 |
| **Osama 2015** | 1 | 12 | 3 | 40.0 | 36.76 | -2.77 | 1.56 | 50 | 0.26 | 1.65 | 50 |
| **Zheng 2015** | 3 | 24 | 4 | 40.0 | 61.5 | -6.10 | 6.44 | 55 | -2.10 | 6.89 | 57 |
| ***Pibernik 2015*** | 1 | 6 | 1 | 90.0 | 58.35 | -1.70 | 7.13 | 58 | -1.60 | 6.83 | 5 |
| ***Leehey 2016*** | 2 | 52 | 4 | 60.0 | 66 | -2.70 | 6.99 | 14 | 1.30 | 9.08 | 18 |
| **Yucel 2016** | 3 | 12 | 3 | 57.5 | 56 | -1.00 | 1.55 | 24 | -1.00 | 1.48 | 21 |
| **Schneider 2016** | 1 | 22 | 2 | 90.0 | 53.45 | -6.00 | 10.79 | 15 | -9.30 | 3.45 | 14 |
| **Delevatti 2018** | 4 | 12 | 3 | 45.0 | 56.7 | -1.20 | 1.14 | 17 | 0.40 | 1.02 | 18 |
| ***Gilani 2019*** | 1 | 12 | 3 | 52.5 | 48.97 | -2.96 | 3.10 | 30 | 0.47 | 3.25 | 30 |
| **Duruturk 2019** | 1 | 6 | 3 | 32.5 | 52.93 | -3.13 | 6.93 | 23 | -0.05 | 6.36 | 21 |
| **Abdelbasset 2020** | 3 | 8 | 3 | 45.0 | 53.1 | -10.80 | 6.09 | 14 | -2.80 | 5.90 | 14 |
| **Singh 2020** | 3 | 12 | 5 | 30.0 | 49.85 | -8.80 | 3.15 | 101 | -4.00 | 3.43 | 99 |
| **Martinez 2021** | 2 | 12 | 6 | 40.0 | 86.5 | -1.50 | 2.37 | 54 | 0.70 | 2.40 | 49 |
| **Maharaj 2023** | 1 | 12 | 3 | 45.0 | 40.15 | -2.70 | 2.31 | 25 | -0.30 | 3.81 | 24 |
| **Donyaei 2024** | 2 | 12 | 3 | 60.0 | 61.7 | -17.47 | 2.42 | 17 | -0.29 | 2.98 | 17 |
| **Delevatti 2025a** | 4 | 15 | 3 | 56.0 | 58.05 | -2.08 | 3.35 | 19 | -2.19 | 3.36 | 18 |
| **Delevatti 2025b** | 2 | 15 | 3 | 56 | 59.75 | -0.28 | 4.82 | 19 | -2.19 | 3.36 | 18 |
| **Elgayar 2025** | 1 | 12 | 3 | 26.0 | 49.25 | -6.63 | 3.70 | 37 | -1.15 | 3.51 | 39 |
| **Subramani 2025** | 3 | 12 |  | 35.0 | 53 | -3.50 | 4.06 | 53 | 1.40 | 2.91 | 70 |
