# Supplementary material for: Effects of different exercise modalities on depressive symptom score changes in patients with type 2 diabetes: a systematic review and network meta-analysis
Source: Front Public Health. 2026 Jul 8;14:1880248. doi: 10.3389/fpubh.2026.1880248 (PMC13393424; doi:10.3389/fpubh.2026.1880248)

**Appendix：**

**Appendix 1.** PRISMA NMA checklist of items to include when reporting a systematic review involving a network meta-analysis.

| **Section/Topic** | **Item #** | **Checklist Item** | **Reported on Page #** |
| --- | --- | --- | --- |
| **TITLE** |  |  |  |
| Title | 1 | Identify the report as a systematic review *incorporating a network meta-analysis (or related form of meta-analysis).* | **P1**  **Lines 1-3** |
|  |  |  |  |
| **ABSTRACT** |  |  |  |
| Structured summary | 2 | Provide a structured summary including, as applicable:  **Background:** main objectives  **Methods:** data sources; study eligibility criteria, participants, and interventions; study appraisal; and *synthesis methods, such as network meta-analysis.*  **Results:** number of studies and participants identified; summary estimates with corresponding confidence/credible intervals; *treatment rankings may also be discussed. Authors may choose to summarize pairwise comparisons against a chosen treatment included in their analyses for brevity.*  **Discussion/Conclusions:** limitations; conclusions and implications of findings.  **Other:** primary source of funding; systematic review registration number with registry name. | **P1-P2**  **Lines 13-44** |
|  |  |  |  |
| **INTRODUCTION** |  |  |  |
| Rationale | 3 | Describe the rationale for the review in the context of what is already known*, including mention of why a network meta-analysis has been conducted.* | **P2-P3**  **Lines 46-98** |
| Objectives | 4 | Provide an explicit statement of questions being addressed, with reference to participants, interventions, comparisons, outcomes, and study design (PICOS). | **P3**  **Lines 90-98** |
|  |  |  |  |
| **METHODS** |  |  |  |
| Protocol and registration | 5 | Indicate whether a review protocol exists and if and where it can be accessed (e.g., Web address); and, if available, provide registration information, including registration number. | **P3**  **Lines 101-104** |
| Eligibility criteria | 6 | Specify study characteristics (e.g., PICOS, length of follow-up) and report characteristics (e.g., years considered, language, publication status) used as criteria for eligibility, giving rationale. *Clearly describe eligible treatments included in the treatment network, and note whether any have been clustered or merged into the same node (with justification).* | **P3-P4**  **Lines 119-130** |
| Information sources | 7 | Describe all information sources (e.g., databases with dates of coverage, contact with study authors to identify additional studies) in the search and date last searched. | **P3**  **Lines 106-110** |
| Search | 8 | Present full electronic search strategy for at least one database, including any limits used, such that it could be repeated. | **Appendix 2** |
| Study selection | 9 | State the process for selecting studies (i.e., screening, eligibility, included in systematic review, and, if applicable, included in the meta-analysis). | **P3**  **Lines 110-117** |
| Data collection process | 10 | Describe method of data extraction from reports (e.g., piloted forms, independently, in duplicate) and any processes for obtaining and confirming data from investigators. | **P5**  **Lines 168-179** |
| Data items | 11 | List and define all variables for which data were sought (e.g., PICOS, funding sources) and any assumptions and simplifications made. | **P3**  **Lines 110-117**  **Table 1** |
| **Geometry of the network** | **S1** | Describe methods used to explore the geometry of the treatment network under study and potential biases related to it. This should include how the evidence base has been graphically summarized for presentation, and what characteristics were compiled and used to describe the evidence base to readers. | **P7**  **Lines 258-263** |
| Risk of bias within individual studies | 12 | Describe methods used for assessing risk of bias of individual studies (including specification of whether this was done at the study or outcome level), and how this information is to be used in any data synthesis. | **P5**  **Lines 181-188** |
| Summary measures | 13 | State the principal summary measures (e.g., risk ratio, difference in means). *Also describe the use of additional summary measures assessed, such as treatment rankings and surface under the cumulative ranking curve (SUCRA) values, as well as modified approaches used to present summary findings from meta-analyses.* | **P7**  **Lines 255-258**  **Lines 276-283** |
| Planned methods of analysis | 14 | Describe the methods of handling data and combining results of studies for each network meta-analysis. This should include, but not be limited to:   - *Handling of multi-arm trials;* - *Selection of variance structure;* - *Selection of prior distributions in Bayesian analyses; and* - *Assessment of model fit.* | **P6-P7**  **Lines 210-238**  **Lines 247-255** |
| **Assessment of Inconsistency** | **S2** | Describe the statistical methods used to evaluate the agreement of direct and indirect evidence in the treatment network(s) studied. Describe efforts taken to address its presence when found. | **P7**  **Lines 264-275** |
| Risk of bias across studies | 15 | Specify any assessment of risk of bias that may affect the cumulative evidence (e.g., publication bias, selective reporting within studies). | **P5**  **Lines 189-208** |
| Additional analyses | 16 | Describe methods of additional analyses if done, indicating which were pre-specified. This may include, but not be limited to, the following:   - Sensitivity or subgroup analyses; - Meta-regression analyses; - *Alternative formulations of the treatment network; and* - *Use of alternative prior distributions for Bayesian analyses (if applicable).* | **P6-P7**  **Lines 238-243**  **Lines 283-289** |
|  |  |  |  |
| **RESULTS†** |  |  |  |
| Study selection | 17 | Give numbers of studies screened, assessed for eligibility, and included in the review, with reasons for exclusions at each stage, ideally with a flow diagram. | **P8-P9**  **Lines 310-314**  **Figure 1** |
| **Presentation of network structure** | **S3** | Provide a network graph of the included studies to enable visualization of the geometry of the treatment network. | **P19**  **Figure 4** |
| **Summary of network geometry** | **S4** | Provide a brief overview of characteristics of the treatment network. This may include commentary on the abundance of trials and randomized patients for the different interventions and pairwise comparisons in the network, gaps of evidence in the treatment network, and potential biases reflected by the network structure. | **P18**  **Lines 402-422** |
| Study characteristics | 18 | For each study, present characteristics for which data were extracted (e.g., study size, PICOS, follow-up period) and provide the citations. | **P9-P15**  **Lines 319-360**  **Table 1** |
| Risk of bias within studies | 19 | Present data on risk of bias of each study and, if available, any outcome level assessment. | **P15-P16**  **Lines 370-396**  **Figure 2**  **Figure 3** |
| Results of individual studies | 20 | For all outcomes considered (benefits or harms), present, for each study: 1) simple summary data for each intervention group, and 2) effect estimates and confidence intervals. *Modified approaches may be needed to deal with information from larger networks.* | **P19**  **Lines 427-435**  **Table 2** |
| Synthesis of results | 21 | Present results of each meta-analysis done, including confidence/credible intervals. *In larger networks, authors may focus on comparisons versus a particular comparator (e.g. placebo or standard care), with full findings presented in an appendix. League tables and forest plots may be considered to summarize pairwise comparisons.* If additional summary measures were explored (such as treatment rankings), these should also be presented. | **P18-P20**  **Lines 402-409**  **Lines 438-446**  **Table 2**  **Table 3 Appendix 3**  **Appendix 5** |
| **Exploration for inconsistency** | **S5** | Describe results from investigations of inconsistency. This may include such information as measures of model fit to compare consistency and inconsistency models, *P* values from statistical tests, or summary of inconsistency estimates from different parts of the treatment network. | **Appendix 4** |
| Risk of bias across studies | 22 | Present results of any assessment of risk of bias across studies for the evidence base being studied. | **P21-P23**  **Lines 472-489**  **Table 4** |
| Results of additional analyses | 23 | Give results of additional analyses, if done (e.g., sensitivity or subgroup analyses, meta-regression analyses*, alternative network geometries studied, alternative choice of prior distributions for Bayesian analyses,* and so forth). | **P20-P21**  **Figure 5**  **Figure 6**  **Appendix 5** |
|  |  |  |  |
| **DISCUSSION** |  |  |  |
| Summary of evidence | 24 | Summarize the main findings, including the strength of evidence for each main outcome; consider their relevance to key groups (e.g., healthcare providers, users, and policy-makers). | **P23-P24**  **Lines 497-556** |
| Limitations | 25 | Discuss limitations at study and outcome level (e.g., risk of bias), and at review level (e.g., incomplete retrieval of identified research, reporting bias). *Comment on the validity of the assumptions, such as transitivity and consistency. Comment on any concerns regarding network geometry (e.g., avoidance of certain comparisons).* | **P24-P25**  **Lines 558-589** |
| Conclusions | 26 | Provide a general interpretation of the results in the context of other evidence, and implications for future research. | **P25-P26**  **Lines 603-636** |
|  |  |  |  |
| **FUNDING** |  |  |  |
| Funding | 27 | Describe sources of funding for the systematic review and other support (e.g., supply of data); role of funders for the systematic review. This should also include information regarding whether funding has been received from manufacturers of treatments in the network and/or whether some of the authors are content experts with professional conflicts of interest that could affect use of treatments in the network. | **P26**  **Lines 654-655** |

PICOS = population, intervention, comparators, outcomes, study design.

* Text in italics indicateS wording specific to reporting of network meta-analyses that has been added to guidance from the PRISMA statement.

† Authors may wish to plan for use of appendices to present all relevant information in full detail for items in this section.

**Appendix 2. Search strategy**

**Database: PubMed**

**Data of Search: April 20, 2026**

| Step | Search Terms | Results |
| --- | --- | --- |
| #1 | "Diabetes Mellitus. Type 2" [Mesh] | 198,692 |
| #2 | ((((((((((((((((((((((((((((((Diabetes Mellitus, Adult-Onset[Title/Abstract]) OR (Adult-Onset Diabetes Mellitus[Title/Abstract])) OR (Diabetes Mellitus, Adult Onset[Title/Abstract])) OR (Diabetes Mellitus, Ketosis-Resistant[Title/Abstract])) OR (Diabetes Mellitus, Ketosis Resistant[Title/Abstract])) OR (Ketosis-Resistant Diabetes Mellitus[Title/Abstract])) OR (NIDDM[Title/Abstract])) OR (Diabetes Mellitus, Maturity-Onset[Title/Abstract])) OR (Diabetes Mellitus, Maturity Onset[Title/Abstract])) OR (Diabetes Mellitus, Non Insulin Dependent[Title/Abstract])) OR (Diabetes Mellitus, Non-Insulin-Dependent[Title/Abstract])) OR (Non-Insulin-Dependent Diabetes Mellitus[Title/Abstract])) OR (Diabetes Mellitus, Noninsulin Dependent[Title/Abstract])) OR (Diabetes Mellitus, Noninsulin-Dependent[Title/Abstract])) OR (Diabetes Mellitus, Slow-Onset[Title/Abstract])) OR (Diabetes Mellitus, Slow Onset[Title/Abstract])) OR (Slow-Onset Diabetes Mellitus[Title/Abstract])) OR (Diabetes Mellitus, Stable[Title/Abstract])) OR (Stable Diabetes Mellitus[Title/Abstract])) OR (Diabetes Mellitus, Type II[Title/Abstract])) OR (Maturity-Onset Diabetes[Title/Abstract])) OR (Diabetes, Maturity-Onset[Title/Abstract])) OR (Maturity Onset Diabetes[Title/Abstract])) OR (Maturity-Onset Diabetes Mellitus[Title/Abstract])) OR (Maturity Onset Diabetes Mellitus[Title/Abstract])) OR (MODY[Title/Abstract])) OR (Noninsulin-Dependent Diabetes Mellitus[Title/Abstract])) OR (Noninsulin Dependent Diabetes Mellitus[Title/Abstract])) OR (Type 2 Diabetes[Title/Abstract])) OR (Diabetes, Type 2[Title/Abstract])) OR (Type 2 Diabetes Mellitus[Title/Abstract]) | 224,045 |
| #3 | #1 OR #2 | 279,662 |
| #4 | "Exercise"[Mesh] | 285,663 |
| #5 | ((((((((((((((((((((((((((((((((Exercises[Title/Abstract]) OR (Exercise, Physical[Title/Abstract])) OR (Exercises, Physical[Title/Abstract])) OR (Physical Exercise[Title/Abstract])) OR (Physical Exercises[Title/Abstract])) OR (Exercise, Isometric[Title/Abstract])) OR (Exercises, Isometric[Title/Abstract])) OR (Isometric Exercises[Title/Abstract])) OR (Isometric Exercise[Title/Abstract])) OR (Exercise, Aerobic[Title/Abstract])) OR (Aerobic Exercise[Title/Abstract])) OR (Aerobic Exercises[Title/Abstract])) OR (Exercises, Aerobic[Title/Abstract])) OR (Exercise Training[Title/Abstract])) OR (Exercise Trainings[Title/Abstract])) OR (Training, Exercise[Title/Abstract])) OR (Trainings, Exercise[Title/Abstract])) OR (Physical Activity[Title/Abstract])) OR (Activities, Physical[Title/Abstract])) OR (Activity, Physical[Title/Abstract])) OR (Physical Activities[Title/Abstract])) OR (Active Breaks[Title/Abstract])) OR (Activity Breaks[Title/Abstract])) OR (Acute Exercise[Title/Abstract])) OR (Acute Exercises[Title/Abstract])) OR (Exercise, Acute[Title/Abstract])) OR (Exercises, Acute[Title/Abstract])) OR (strength training[Title/Abstract])) OR (yoga[Title/Abstract])) OR (tai chi[Title/Abstract])) OR (qigong[Title/Abstract])) OR (intenvent*[Title/Abstract])) OR (resistance training[Title/Abstract]) | 382,480 |
| #6 | #4 OR #5 | 522,806 |
| #7 | "Depression"[Mesh] | 180,179 |
| #8 | (((((((melancholia[Title/Abstract]) OR (mental health[Title/Abstract])) OR (psychological health[Title/Abstract])) OR (Depressive Symptoms[Title/Abstract])) OR (Depressive Symptom[Title/Abstract])) OR (Symptom, Depressive[Title/Abstract])) OR (Emotional Depression[Title/Abstract])) OR (Depression, Emotional[Title/Abstract]) | 389,167 |
| #9 | #7 OR #8 | 506,372 |
| #10 | ((randomized controlled trial[Title/Abstract]) OR (randomized[Title/Abstract])) OR (placebo[Title/Abstract]) | 1,835,869 |
| #11 | #3 AND #6 AND #9 AND #10 | 169 |

**Database: Web Of Science**

**Data of Search: April 20, 2026**

| Step | Search Terms | Results |
| --- | --- | --- |
| #1 | TS=('Diabetes Mellitus, Adult-Onset' or 'Adult-Onset Diabetes Mellitus' or 'Diabetes Mellitus, Adult Onset' or 'Diabetes Mellitus, Ketosis-Resistant' or 'Diabetes Mellitus, Ketosis Resistant' or 'Ketosis-Resistant Diabetes Mellitus' or 'NIDDM' or 'Diabetes Mellitus, Maturity-Onset' or 'Diabetes Mellitus, Maturity Onset' or 'Diabetes Mellitus, Non Insulin Dependent' or 'Diabetes Mellitus, Non-Insulin-Dependent' or 'Non-Insulin-Dependent Diabetes Mellitus' or 'Diabetes Mellitus, Noninsulin Dependent' or 'Diabetes Mellitus, Noninsulin-Dependent' or 'Diabetes Mellitus, Slow-Onset' or 'Diabetes Mellitus, Slow Onset' or 'Slow-Onset Diabetes Mellitus' or 'Diabetes Mellitus, Stable' or 'Stable Diabetes Mellitus' or 'Diabetes Mellitus, Type II' or 'Maturity-Onset Diabetes' or 'Maturity Onset Diabetes' or 'Diabetes, Maturity-Onset' or 'Maturity-Onset Diabetes Mellitus' or 'Maturity Onset Diabetes Mellitus' or 'MODY ' or 'Noninsulin-Dependent Diabetes Mellitus' or 'Noninsulin Dependent Diabetes Mellitus' or 'Type 2 Diabetes' or 'Diabetes, Type 2' or 'Type 2 Diabetes Mellitus' or 'Diabetes Mellitus, Type 2') | 268,965 |
| #2 | TS=('Exercise' or 'Exercises' or 'Exercise, Physical' or 'Exercises, Physical' or 'Physical Exercise' or 'Physical Exercises' or 'Exercise, Isometric' or 'Exercises, Isometric' or 'Isometric Exercises' or 'Isometric Exercise' or 'Exercise, Aerobic' or 'Aerobic Exercise' or 'Aerobic Exercises' or 'Exercises, Aerobic' or 'Exercise Training' or 'Exercise Trainings' or 'Training, Exercise' or 'Trainings, Exercise' or 'Physical Activity' or 'Activities, Physical' or 'Activity, Physical' or 'Physical Activities' or 'Active Breaks' or 'Activity Breaks' or 'Acute Exercise' or 'Acute Exercises' or 'Exercise, Acute' or 'Exercises, Acute' or 'strength training' or 'yoga' or 'tai chi' or 'qigong' or 'intenvent*' or 'resistance training') | 828,926 |
| #3 | TS=('depression' or 'melancholia' or 'mental health' or 'psychological health' or 'Depressive Symptoms' or 'Depressive Symptom' or 'Symptom, Depressive' or 'Emotional Depression' or 'Depression, Emotional') | 943,950 |
| #4 | TS=('randomized controlled trial' or 'randomized' or 'placebo') | 1,060,765 |
| #5 | #1 AND #2 AND #3 AND #4 | 449 |

**Database: Cochrane Library**

**Data of Search: April 20, 2026**

| Step | Search Terms | Results |
| --- | --- | --- |
| #1 | MeSH descriptor: [Diabetes Mellitus, Type 2] explode all trees | 27,733 |
| #2 | (Diabetes Mellitus, Adult-Onset):ti,ab,kw or (Adult-Onset Diabetes Mellitus):ti,ab,kw or (Diabetes Mellitus, Adult Onset):ti,ab,kw or (Diabetes Mellitus, Ketosis-Resistant):ti,ab,kw or (Diabetes Mellitus, Ketosis Resistant):ti,ab,kw or (Ketosis-Resistant Diabetes Mellitus):ti,ab,kw or (NIDDM):ti,ab,kw or (Diabetes Mellitus, Maturity-Onset):ti,ab,kw or (Diabetes Mellitus, Maturity Onset):ti,ab,kw or (Diabetes Mellitus, Non Insulin Dependent):ti,ab,kw or (Diabetes Mellitus, Non-Insulin-Dependent):ti,ab,kw or (Non-Insulin-Dependent Diabetes Mellitus):ti,ab,kw or (Diabetes Mellitus, Noninsulin Dependent):ti,ab,kw or (Diabetes Mellitus, Noninsulin-Dependent):ti,ab,kw or (Diabetes Mellitus, Slow-Onset):ti,ab,kw or (Diabetes Mellitus, Slow Onset):ti,ab,kw or (Slow-Onset Diabetes Mellitus):ti,ab,kw or (Diabetes Mellitus, Stable):ti,ab,kw or (Stable Diabetes Mellitus):ti,ab,kw or (Diabetes Mellitus, Type II):ti,ab,kw or (Maturity-Onset Diabetes):ti,ab,kw or (Diabetes, Maturity-Onset):ti,ab,kw or (Maturity Onset Diabetes):ti,ab,kw or (Maturity-Onset Diabetes Mellitus):ti,ab,kw or (Maturity Onset Diabetes Mellitus):ti,ab,kw or (MODY ):ti,ab,kw or (Noninsulin-Dependent Diabetes Mellitus):ti,ab,kw or (Noninsulin Dependent Diabetes Mellitus):ti,ab,kw or (Type 2 Diabetes):ti,ab,kw or (Diabetes, Type 2):ti,ab,kw or (Type 2 Diabetes Mellitus):ti,ab,kw | 73,205 |
| #3 | #1 or #2 | 73,206 |
| #4 | MeSH descriptor: [Exercise] explode all trees | 42,845 |
| #5 | (Exercises):ti,ab,kw or (Exercise, Physical):ti,ab,kw or (Exercises, Physical):ti,ab,kw or (Physical Exercise):ti,ab,kw or (Physical Exercises):ti,ab,kw or (Exercise, Isometric):ti,ab,kw or (Exercises, Isometric):ti,ab,kw or (Isometric Exercises):ti,ab,kw or (Isometric Exercise):ti,ab,kw or (Exercise, Aerobic):ti,ab,kw or (Aerobic Exercise):ti,ab,kw or (Aerobic Exercises):ti,ab,kw or (Exercises, Aerobic):ti,ab,kw or (Exercise Training):ti,ab,kw or (Exercise Trainings):ti,ab,kw or (Training, Exercise):ti,ab,kw or (Trainings, Exercise):ti,ab,kw or (Physical Activity):ti,ab,kw or (Activities, Physical):ti,ab,kw or (Activity, Physical):ti,ab,kw or (Physical Activities):ti,ab,kw or (Active Breaks):ti,ab,kw or (Activity Breaks):ti,ab,kw or (Acute Exercise):ti,ab,kw or (Acute Exercises):ti,ab,kw or (Exercise, Acute):ti,ab,kw or (Exercises, Acute):ti,ab,kw or (strength training):ti,ab,kw or (yoga):ti,ab,kw or (tai chi):ti,ab,kw or (qigong):ti,ab,kw or (intenvent*):ti,ab,kw or (resistance training):ti,ab,kw | 181,777 |
| #6 | #4 or #5 | 191,009 |
| #7 | MeSH descriptor: [Depression] explode all trees | 20,305 |
| #8 | (melancholia):ti, ab,kw or (mental health):ti,ab,kw or (psychological health):ti, ab,kw or (Depressive Symptoms):ti, ab,kw or (Depressive Symptom):ti, ab,kw or (Symptom, Depressive):ti,ab,kw or (Emotional Depression):ti,ab,kw or (Depression, Emotional):ti,ab,kw | 101,543 |
| #9 | #7 or #8 | 110,575 |
| #10 | #3 and #6 and #9 | 901 |

**Database: Embase**

**Data of Search: April 20, 2026**

| Step | Search Terms | Results |
| --- | --- | --- |
| #1 | 'non insulin dependent diabetes mellitus'/exp | 458,324 |
| #2 | diabetes mellitus, adult-onset':ab,ti OR 'adult-onset diabetes mellitus':ab,ti OR 'diabetes mellitus, adult onset':ab,ti OR 'diabetes mellitus, ketosis-resistant':ab,ti OR 'diabetes mellitus, ketosis resistant':ab, ti OR 'ketosis-resistant diabetes mellitus':ab,ti OR 'niddm':ab,ti OR 'diabetes mellitus, maturity-onset':ab,ti OR 'diabetes mellitus, maturity onset':ab,ti OR 'diabetes mellitus, non insulin dependent':ab, ti OR 'diabetes mellitus, non-insulin-dependent':ab,ti OR 'non-insulin-dependent diabetes mellitus':ab,ti OR 'diabetes mellitus, noninsulin dependent':ab,ti OR 'diabetes mellitus, noninsulin-dependent':ab,ti OR 'diabetes mellitu ow-onset':ab,ti OR 'diabetes mellitus, slow onset':ab, ti OR 'slow-onset diabetes mellitus':ab,ti OR 'diabetes mellitus, stable':ab,ti OR 'stable diabetes mellitus':ab, ti OR 'diabetes mellitus, type ':ab,ti OR 'maturity-onset diabetes':ab,ti OR 'diabetes, maturity-onset':ab, ti OR 'maturity onset diabetes':ab, ti OR 'maturity-onset diabetes mellitus':ab, ti OR 'maturity onset diabetes mellitus':ab, ti OR 'mody":ab, ti OR 'noninsulin-dependent diabetes mellitus':ab,ti OR 'noninsulin dependent diabetes mellitus':ab, ti OR 'type 2 diabetes':ab,ti OR 'diabetes, type 2':ab, ti OR 'type 2 diabetes mellitus':ab,ti | 443,906 |
| #3 | #1 OR #2 | 586,983 |
| #4 | 'exercise'/exp | 576,655 |
| #5 | exercises:ab,ti OR (exercise,:ab,ti AND physical:ab,ti) OR (exercises,:ab,ti AND physical:ab,ti) OR physical exercise:ab,ti OR (physical:ab,ti AND exercises:ab,ti) OR (exercise,:ab, ti AND isometric:ab,ti) OR (exercises,:ab,ti AND isometric:ab,ti) OR (isometric:ab,ti AND exercises:ab,ti) OR isometric exercise:ab,ti OR (exercise,:ab,ti AND aerobic:ab,ti) OR aerobic exercise:ab,ti OR (aerobic:ab,ti AND exercises:ab,ti) OR (exercises, ab, ti AND aerobic:ab, ti) OR exercise training:ab,ti OR exercise trainings:ab,ti OR training, exercise:ab,ti OR trainings, exercise:ab,ti OR (physical:ab,ti AND activity:ab,ti) OR (activities,:ab, ti AND physical:ab,ti) OR (activity,:ab,ti AND physical:ab,ti) OR (physical:ab,ti AND activities:ab,ti) OR (active:ab,ti AND breaks:ab,ti) OR (activity:ab,ti AND breaks:ab,ti) OR acute exercise:ab, ti OR (acute:ab,ti AND exercises:ab,ti) OR (exercise,:ab,ti AND acute:ab, ti) OR (exercises,:ab,ti AND acute:ab,ti) OR (strength:ab,ti AND training:ab,ti) OR yoga:ab,ti OR (tai:ab,ti AND chi:ab,ti) OR qigong:ab, ti OR intenvent*:ab,ti OR (resistance:ab,ti AND training:ab,ti) | 721,474 |
| #6 | #4 OR #5 | 1,015,772 |
| #7 | 'depression'/exp | 829,374 |
| #8 | melancholia:ab,ti OR (mental:ab,ti AND health:ab,ti) OR (psychological:ab,ti AND health:ab,ti) OR (depressive:ab,ti AND symptoms:ab,ti) OR (depressive:ab,ti AND symptom:ab,ti) OR (symptom,:ab,ti AND depressive:ab,ti) OR emotional depression:ab,ti OR (depression,:ab,ti AND emotional:ab,ti) | 717,792 |
| #9 | #7 OR #8 | 1,277,327 |
| #10 | 'randomized controlled trial':ab,ti OR 'randomized" :ab,ti OR 'placebo':ab, ti | 1,574,156 |
| #11 | #3 AND #6 AND #9 AND #10 | 738 |

**Database: Ebsco**

**Data of Search: April 20, 2026**

| Step | Search Terms | Results |
| --- | --- | --- |
| #1 | AB Diabetes Mellitus, Adult-Onset OR AB Adult-Onset Diabetes Mellitus OR AB Diabetes Mellitus, Adult Onset OR AB Diabetes Mellitus, Ketosis-Resistant OR AB Diabetes Mellitus, Ketosis Resistant OR AB Ketosis-Resistant Diabetes Mellitus OR AB NIDDM OR AB Diabetes Mellitus, Maturity-Onset OR AB Diabetes Mellitus, Maturity Onset OR AB Diabetes Mellitus, Non Insulin Dependent OR AB Diabetes Mellitus, Non-Insulin-Dependent OR AB Non-Insulin-Dependent Diabetes Mellitus OR AB Diabetes Mellitus, Noninsulin Dependent OR AB Diabetes Mellitus, Noninsulin-Dependent OR AB Diabetes Mellitus, Slow-Onset OR AB Diabetes Mellitus, Slow Onset OR AB Slow-Onset Diabetes Mellitus OR AB Diabetes Mellitus, Stable OR AB Stable Diabetes Mellitus OR AB Diabetes Mellitus, Type II OR AB Maturity-Onset Diabetes OR AB Diabetes, Maturity-Onset OR AB Maturity Onset Diabetes OR AB Maturity-Onset Diabetes Mellitus OR AB Maturity Onset Diabetes Mellitus OR AB MODY OR AB Noninsulin-Dependent Diabetes Mellitus OR AB Noninsulin Dependent Diabetes Mellitus OR AB Type 2 Diabetes OR AB Diabetes, Type 2 OR AB Type 2 Diabetes Mellitus Diabetes Mellitus, Type 2 |  |
| #2 | AB Exercise OR AB Exercises OR AB Exercise, Physical OR AB Exercises, Physical OR AB Physical Exercise OR AB Physical Exercises OR AB Exercise, Isometric OR AB Exercises, Isometric OR AB Isometric Exercises OR AB Isometric Exercise OR AB Exercise, Aerobic OR AB Aerobic Exercise OR AB Aerobic Exercises OR AB Exercises, Aerobic OR AB Exercise Training OR AB Exercise Trainings OR AB Training, Exercise OR AB Trainings, Exercise OR AB Physical Activity OR AB Activities, Physical OR AB Activity, Physical OR AB Physical Activities OR AB Active Breaks OR AB Activity Breaks OR AB Acute Exercise OR AB Acute Exercises Exercise, Acute OR AB Exercises, Acute OR AB strength training OR AB yoga OR AB tai chi OR AB qigong OR AB intenvent* OR AB resistance training |  |
| #3 | AB depression OR AB melancholia OR AB mental health OR AB psychological health OR AB Depressive Symptoms OR AB Depressive Symptom OR AB Symptom, Depressive OR AB Emotional Depression OR AB Depression, Emotional |  |
| #4 | AB randomized controlled trial OR AB randomized OR AB placebo |  |
|  | #1 AND #2 AND #3 AND #4 | 181 |

**Appendix 3.** The direct and indirect contributions of non-inferiority comparison analysis (NMA) and the number of studies for each direct comparison were examined.

**Note:** A-CON, B-AE, C-COMB, D-MBE, E-AT

**
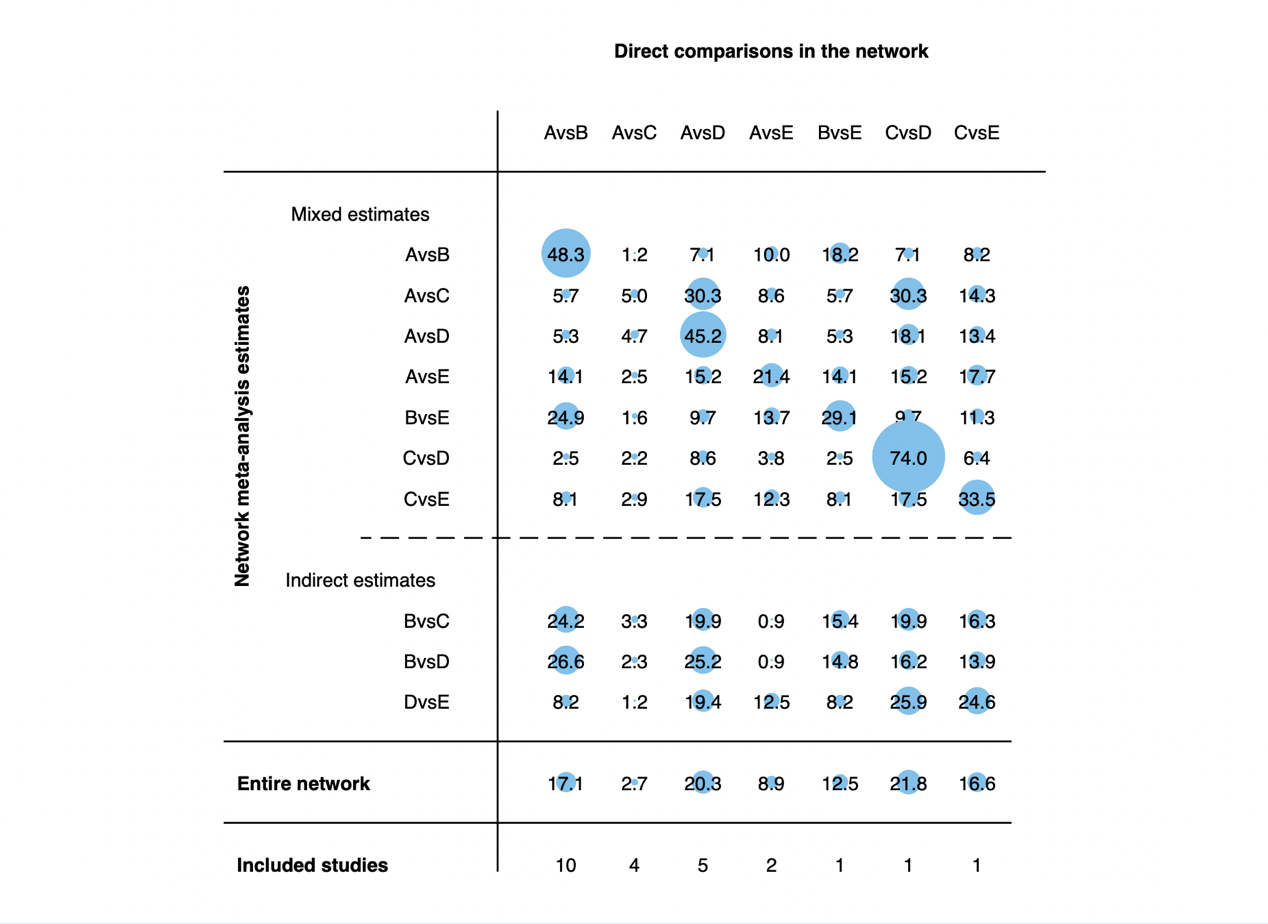
**

**Appendix 4.** The inconsistency was tested through the estimation of heterogeneity in specific circuits, the inconsistent models, and the analysis of node segmentation.

**Note:** A-CON, B-AE, C-COMB, D-MBE, E-AT

| **Loop** | **IF** | **seIF** | **z_value** | **p_value** | **CI_95** | **Loop_Heterog_tau2** |
| --- | --- | --- | --- | --- | --- | --- |
| CON-COMB-AT | 2.222 | 3.188 | 0.697 | 0.486 | (0.00,8.47) | 1.563 |
| CON-COMB-MBE | 1.932 | 1.956 | 0.988 | 0.323 | (0.00,5.77) | 0.816 |
| CON-AE-AT | 1.554 | 1.196 | 1.300 | 0.194 | (0.00,3.90) | 0.768 |

| **Inconsistency model** | | |
| --- | --- | --- |
| chi2(4) | = | 6.63 |
| Prob> chi2 | = | 0.1571 |

| **Node splitting analysis** | | | | | | | |
| --- | --- | --- | --- | --- | --- | --- | --- |
| **Side** | **Direct** |  | **Indirect** |  | **Difference** |  |  |
|  | **Coef.** | **Std. Err.** | **Coef.** | **Std. Err.** | **Coef.** | **Std. Err.** | **P>z** |
| A B | -0.483568 | 0.3705562 | 0.7098053 | 1.435794 | -1.193373 | 1.482853 | 0.421 |
| A C | -1.390599 | 0.6190171 | 0.1428591 | 1.131682 | -1.533458 | 1.288941 | 0.234 |
| A D | -0.789935 | 0.5051015 | -2.865761 | 1.246548 | 2.075826 | 1.34492 | 0.123 |
| A E | -0.3740328 | 0.7995737 | -2.313377 | 1.073449 | 1.939344 | 1.338859 | 0.147 |
| B E | -1.446578 | 1.201366 | -0.2531078 | 0.8693525 | -1.19347 | 1.48292 | 0.421 |
| C D | -1.452707 | 1.100196 | 0.6232514 | 0.7736245 | -2.075959 | 1.344963 | 0.123 |
| C E | -0.4519949 | 1.21557 | 0.3341103 | 1.084314 | -0.7861052 | 1.629034 | 0.629 |

**Appendix 5.** The Forest plot comparison.


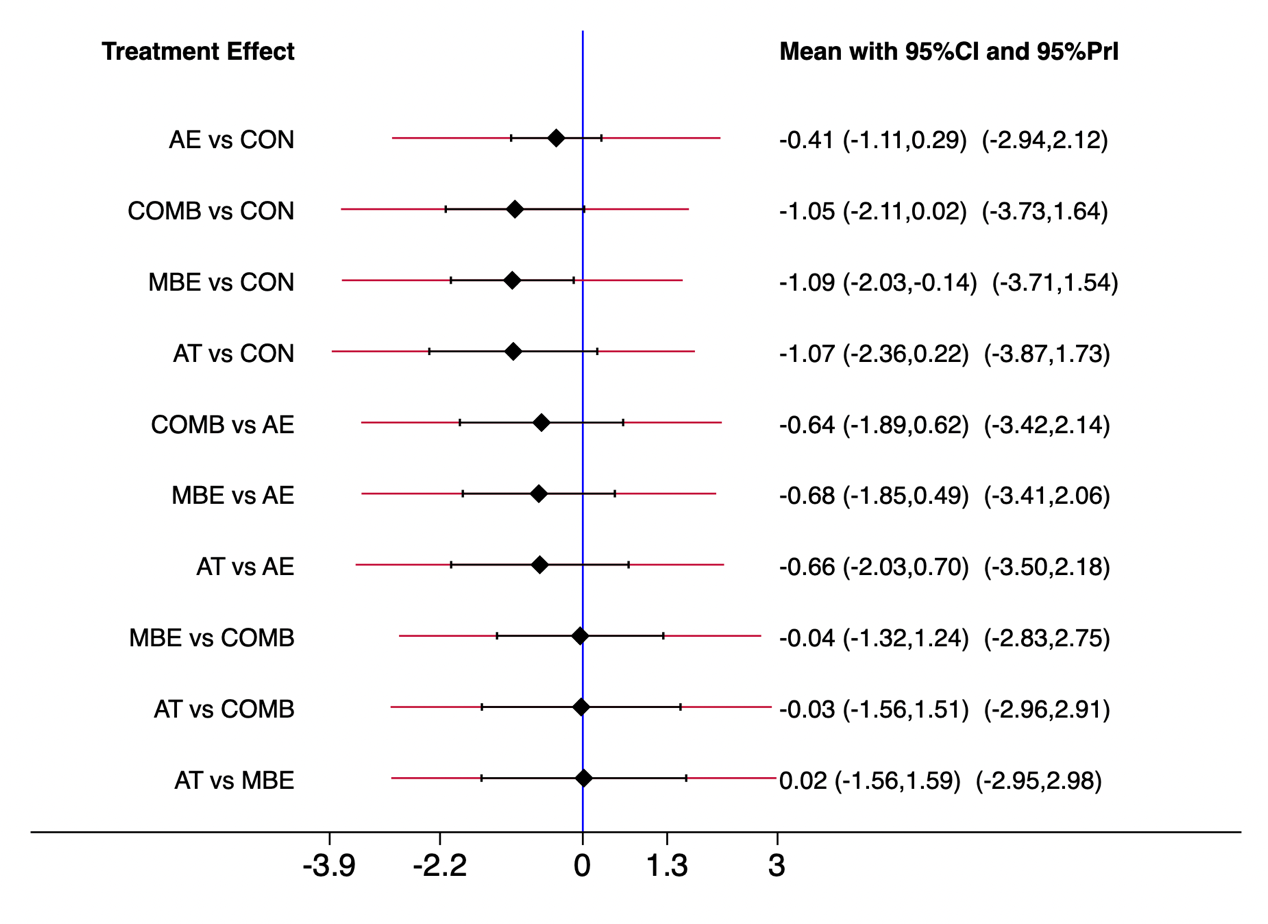


**Appendix 6.** The area under the curve of the cumulative ranking probabilities of each intervention measure for depressive symptoms.


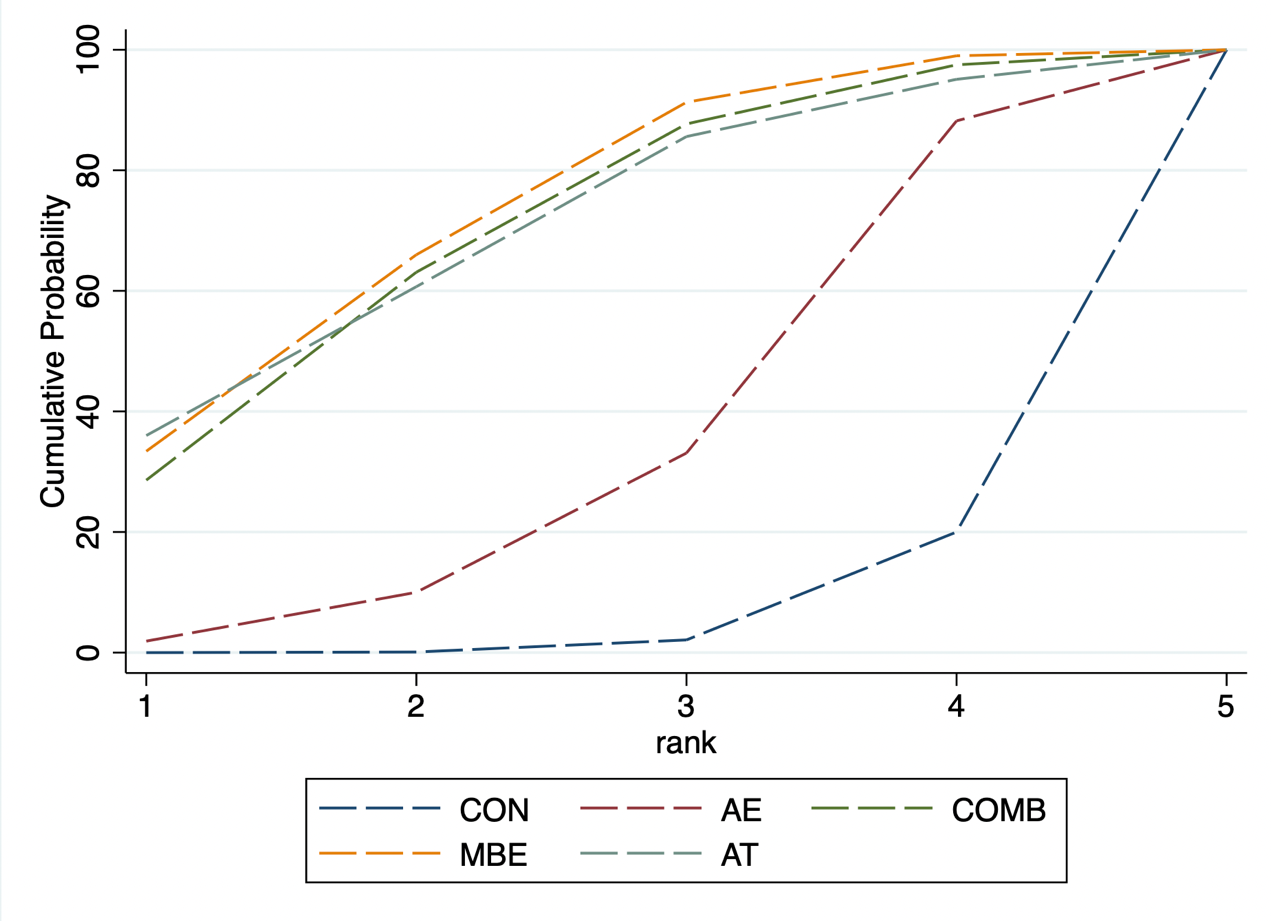

Supplement: Supplementary file 2 [file Table_2.DOCX]
